# Surfactant-assisted one-pot sample preparation for label-free single-cell proteomics

**DOI:** 10.1038/s42003-021-01797-9

**Published:** 2021-03-01

**Authors:** Chia-Feng Tsai, Pengfei Zhang, David Scholten, Kendall Martin, Yi-Ting Wang, Rui Zhao, William B. Chrisler, Dhwani B. Patel, Maowei Dou, Yuzhi Jia, Carolina Reduzzi, Xia Liu, Ronald J. Moore, Kristin E. Burnum-Johnson, Miao-Hsia Lin, Chuan-Chih Hsu, Jon M. Jacobs, Jacob Kagan, Sudhir Srivastava, Karin D. Rodland, H. Steven Wiley, Wei-Jun Qian, Richard D. Smith, Ying Zhu, Massimo Cristofanilli, Tao Liu, Huiping Liu, Tujin Shi

**Affiliations:** 1 Biological Sciences Division, Pacific Northwest National Laboratory, Richland, WA USA; 2 NHC Key Laboratory of Cancer Proteomics, Department of Oncology, Xiangya Hospital, Central South University, Changsha, Hunan P.R. China; 3 Department of Pharmacology, Feinberg School of Medicine, Northwestern University, Chicago, IL USA; 4 Environmental Molecular Sciences Laboratory, Pacific Northwest National Laboratory, Richland, WA USA; 5 Division of Hematology and Oncology, Department of Medicine, Feinberg School of Medicine, Northwestern University, Chicago, IL USA; 6 Graduate Institute of Microbiology, College of Medicine, National Taiwan University, Taipei, Taiwan; 7 Institute of Plant and Microbial Biology, Academia Sinica, Taipei, Taiwan; 8 Cancer Biomarkers Research Group, Division of Cancer Prevention, National Cancer Institute, Bethesda, MD USA; 9 Robert H. Lurie Comprehensive Cancer Center, Feinberg School of Medicine, Northwestern University, Chicago, IL USA

**Keywords:** Proteomic analysis, Proteomics

## Abstract

Large numbers of cells are generally required for quantitative global proteome profiling due to surface adsorption losses associated with sample processing. Such bulk measurement obscures important cell-to-cell variability (cell heterogeneity) and makes proteomic profiling impossible for rare cell populations (e.g., circulating tumor cells (CTCs)). Here we report a surfactant-assisted one-pot sample preparation coupled with mass spectrometry (MS) method termed SOP-MS for label-free global single-cell proteomics. SOP-MS capitalizes on the combination of a MS-compatible nonionic surfactant, n-Dodecyl-β-D-maltoside, and hydrophobic surface-based low-bind tubes or multi-well plates for ‘all-in-one’ one-pot sample preparation. This ‘all-in-one’ method including elimination of all sample transfer steps maximally reduces surface adsorption losses for effective processing of single cells, thus improving detection sensitivity for single-cell proteomics. This method allows convenient label-free quantification of hundreds of proteins from single human cells and ~1200 proteins from small tissue sections (close to ~20 cells). When applied to a patient CTC-derived xenograft (PCDX) model at the single-cell resolution, SOP-MS can reveal distinct protein signatures between primary tumor cells and early metastatic lung cells, which are related to the selection pressure of anti-tumor immunity during breast cancer metastasis. The approach paves the way for routine, precise, quantitative single-cell proteomics.

## Abstract (web summary)

Tsai, Zhang, Scholten et al. develop a surfactant- assisted one-pot sample preparation coupled with mass spectrometry method (SOP-MS) for label-free global single-cell proteomics. This method allows researchers to measure hundreds of proteins from single human cells, suggesting its utility for quantitative single-cell proteomics.

## Introduction

Recent advances in nucleic acid amplification-based sequencing technologies allow for comprehensive characterization of genome and transcriptome in single mammalian or tumor cells^1–3^. Since no protein amplification methods exist for single-cell proteome profiling, current single-cell proteomics technologies primarily rely on antibody-based immunoassays (e.g., mass cytometry) for targeted measurements^4^, but they share the limitations of antibody-based approaches^5^. Mass spectrometry (MS)-based proteomics is a promising alternative for quantitative single-cell proteomics because it is antibody-free and has high specificity and ultrahigh multiplexing capability^6^. Sophisticated sample preparation methods are generally used to process standard proteomics samples with large amounts of starting materials (e.g., ≥1000 µg or ≥10 million human cells) for comprehensive proteomic analysis^7–10^. However, they cannot be used to process smaller samples (e.g., low µg or sub-µg levels of starting materials). With this recognition, in the past decade great efforts have been made for effective processing of smaller samples using single-pot sample preparation (e.g., in-StageTip^11,12^ and SP3^13,14^) and immobilized enzyme processing systems (e.g., IMER^15,16^ and SNaPP^17^). Using the in-StageTip device combined with Tip-based sample fractionation, >7000 proteins across 12 immune cell types were reported when ~15,000 immune cells (~2 µg) were analyzed^12^. The SP3 protocol can allow reproducible quantification of 500-1000 proteins from 100-1000 HeLa cells^14^. With improved sample processing as well as recent advances in detection sensitivity, MS-based single-cell proteomics has recently been used for deep proteome profiling of large-size single cells (e.g., oocytes and blastomeres at ~0.1-100 µg of protein amount per cell)^13,18–20^. However, single-cell proteomic analysis of regular-size mammalian cells (typically ~100 pg per cell) remains highly challenging, primarily due to technical difficulties in effective sampling and processing^21–23^. In recent three years great progress has been made to improve processing recovery from low numbers of cells by either reducing sample processing volume (e.g., nanoPOTS, OAD, and iPAD-1 devices downscaling the processing volume to ~2-200 nL for label-free global proteomics^21,24,25^) or using excessive amounts of carrier proteins or proteome (e.g., the addition of exogenous BSA as a carrier protein for targeted proteomics^22,23^ or tandem mass tag (TMT)-labeled 100s of cells as a carrier channel for TMT labeling-based global proteomics^26^). However, all these approaches have technical drawbacks: nanoPOTS, OAD, and iPAD-1 are not easily adoptable for broad benchtop applications^21,24,25^; exogenous protein carrier is more suitable for targeted proteomics. Peptides from excessive exogenous proteins are frequently sequenced by MS/MS, which greatly reduces the chance for sequencing low abundant endogenous peptides^22,23^; a TMT carrier is added after sample processing, and thus it cannot effectively prevent the surface adsorption losses during initial sample processing^26^, resulting in low reproducibility with a correlation coefficient of only ~0.2–0.4 between replicates for ineffectively processed single cells^27^. Furthermore, due to the inability to fractionate ultrasmall TMT carrier samples, TMT labeling-based global proteomics suffers from ratio compression or distortion caused by coeluting interferences^28^. Therefore, only three MS-based single-cell proteomics methods are available for reliable label-free analysis of regular-size single mammalian cells, but they need specific devices and/or a skilled person to operate which limits their potential for wide adoptions by research community.

Single-cell proteomics can empower characterization of cell functional heterogeneity and reveal important protein signatures at the single-cell level for rare cell populations, such as cancer stem cells, circulating tumor cells (CTCs), and early metastatic cells. When compared to peripheral blood mononuclear cells (PBMCs), CTCs are rare (normally less than 0.1%). Their seeding efficiency is extremely low but CTCs with stem cell properties can cluster and colonize at relatively high efficiency^29–33^. CTCs can remain in the blood stream for up to several hours as single cells or tumor clusters, and sometimes they associate with various other cell types (e.g., neutrophils) until they extravasate at a potential site of metastasis^29,34–36^. However, there are no available tools for proteomic characterization of CTCs that can elucidate their heterogeneity as well as dynamic alterations upon the formation of early micrometastases. Therefore, it still remains uncertain whether metastatic tumor cells undergo an epithelial to mesenchymal transition (EMT) and/or a mesenchymal-to-epithelial transition (MET) at metastatic seeding^37–40^.

**Fig. 1:**
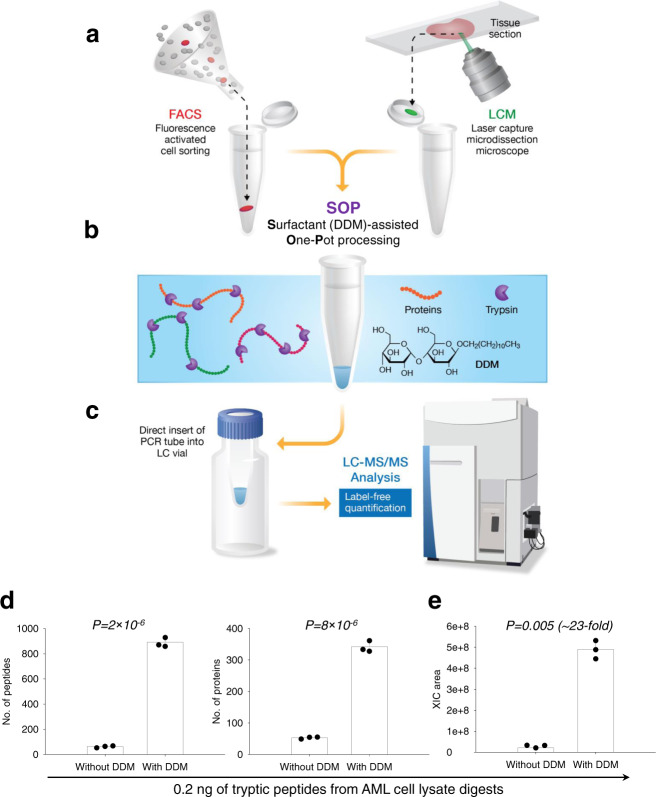
Schematic diagram of the SOP-MS workflow. **a** Single cells or small numbers of cells are sorted either by fluorescence-activated cell sorting (FACS) or laser capture microdissection (LCM) and collected into single PCR tube or a 96-well PCR plate. After FACS isolation, the sorted cells are subjected to centrifugation at 1000*g* for 10 min to ensure them at the bottom of the PCR tube or 96-well PCR plate. For LCM, the dissected tissue voxels are catapulted into a 5 µL water droplet on the PCR tube cap, followed by centrifugation at 1000*g* for 10 min. **b** For cell lysis, a cell lysis buffer containing 0.2% (w/v) n-Dodecyl β-D-maltoside (DDM) is added to the PCR tube or 96-well PCR plate followed by incubation at 75 °C for 1 h. Sample is subjected to reduction and alkylation (these two steps are optional). Small amounts of trypsin are used for overnight digestion: 2 ng for single cells and 5 ng for 10–100 cells, and the final DDM concentration is ~0.015%. **c** Prior to LC-MS analysis, the cap of the PCR tube is removed and the tube is inserted into a sample vial to avoid transfer loss. The 96-well cap matt is used to cover the 96-well plate for automatic injection without sample transfer. Samples are analyzed by standard LC-MS platforms for quantitative proteomic analysis. The freely available open-source MaxQuant software is used for label-free quantification. **d** Number of unique peptides and protein groups identified by MS/MS only for 0.2 ng of tryptic peptides from AML cell lysate digests (three biological replicates per condition) without and with 0.015% DDM (*P* < 0.05 between without and with DDM). **e** The total extracted ion chromatogram (XIC) peak area for 0.2 ng of AML cell lysate digests (three biological replicates per condition) without and with 0.015% DDM (*P* < 0.05 between without and with DDM).

To alleviate the shortcomings of existing proteomic approaches, we have recently developed a broadly adoptable MS method for quantitative label-free single-cell proteomic analysis. This method capitalizes on surfactant-assisted one-pot (single tube or multi-well plate) processing coupled with MS (termed SOP-MS) for greatly reducing the surface adsorption losses, thus improving detection sensitivity for MS analysis of single cells and mass-limited clinical specimens (Fig. 1). SOP-MS was demonstrated to enable reliable label-free quantification of hundreds of proteins from single cells with standard MS platforms. We applied it to analyze two types of single cells isolated from patient CTC-derived xenografts (PCDX): CTCs propagated in the mouse mammary fat pads with CSC properties (primary tumor cells) and their early micrometastases seeded to the lungs (lung micromets). SOP-MS allows not only for the identification of protein signatures from the two different cell types, but also for the elucidation of dynamic alterations of metastatic tumor cells upon colonization of the lungs. Interestingly, many of the altered proteins in the lung metastasis are related to the selection pressure of anti-tumor immunity (e.g., neutrophils and innate immunity) for the transition from primary tumor CTCs to the early metastatic cells. These results demonstrate great potential of SOP-MS for broad applications in the biomedical research.

## Results

### ‘All-in-one’ SOP-MS for maximizing single-cell recovery

The major issue for current MS-based bottom-up single-cell proteomics is substantial surface adsorption losses. Proteins are ‘stickier’ than other biomolecules (e.g., nucleic acids) and need to be digested into peptides for efficient MS analysis which involves multistep sample processing. Both BSA and surfactants are commonly used as additives to minimize surface adsorption for low amounts of proteins and peptides. Unfortunately, the addition of BSA is not suitable for label-free single-cell global proteomics analysis^22,23^. Most ionic surfactants (e.g., sodium dodecyl sulfate) are not MS-compatible and require multiple cleanup steps that cause substantial sample loss, especially for small numbers of cells, though they are highly efficient for cell lysis and protein denaturation^41^. Nonionic surfactants are known to substantially reduce protein adsorption for hydrophobic surface-based vessels (e.g., single tube or single well) while they have less effects on hydrophilic surfaces (e.g., glass vials), because they have much stronger binding strength than proteins for the hydrophobic surface. They are broadly used to modulate protein aggregation, adsorption loss, stability, and activity in the pharmaceutical and biotechnology industries. However, most nonionic surfactants (e.g., octylglucoside) are coeluted with tryptic peptides, which severely affects peptide detection due to ionization suppression^42^.

n-Dodecyl β-D-maltoside (DDM), a classic nonionic surfactant, is an exception. It has been demonstrated to robustly solubilize membrane proteins for effective cell lysis^43,44^, and to be highly compatible with MS without requiring surfactant removal and is eluted at a high percentage of organic solvent where it does not impact peptide detection^43,44^. Furthermore, DDM is sufficiently thermostable to tolerate the high temperature used for cell lysis and protein denaturation, and can also enhance trypsin and Lys-C enzyme activity^42^. Therefore, we have recently developed a nonionic surfactant DDM-assisted one-pot sample preparation coupled with MS termed SOP-MS that combines all steps into one pot (e.g., single PCR tube or single well from a multi-well PCR plate routinely used for single-cell genomics and transcriptomics) including single-cell collection, multistep single-cell processing, and elimination of all transfer steps with direct sample loading for LC-MS analysis (Fig. 1a–c). This ‘all-in-one’ SOP-MS method presumably maximizes single-cell recovery for quantitative single-cell proteomics by greatly reducing possible surface adsorption losses.

To reliably evaluate the performance of SOP-MS, label-free MS was used for proteomic analysis of one cell at a time and protein identification is solely based on the actual MS/MS spectra from the analyzed cell, which is the cornerstone of MS-based proteomics. Furthermore, once it works for label-free MS analysis, SOP-MS can be widely used for other types of MS analysis of single cells. A commonly accessible Q Exactive Plus MS platform was used for the development of SOP-MS and its application demonstration.

### Evaluation of SOP-MS performance using peptides and low-input human cell lysates

To achieve precise proteome quantification of single cells we systematically evaluated sample recovery and processing reproducibility using more uniform low-input (small) samples (i.e., cell lysates or protein digests) with and without DDM in single PCR tubes. Selected reaction monitoring (SRM)-based targeted proteomics was used to optimize DDM concentrations from 0.005% to 0.1% due to its demonstrated higher reproducibility and quantitation accuracy when compared to global proteomics. Heavy isotope-labeled EGFR pathway peptide standards at a fixed concentration were measured at different DDM concentrations. The best SRM signals for most EGFR pathway peptides was achieved with 0.01–0.02% DDM (Supplementary Data 1), where higher DDM concentration can saturate the LC column and thus greatly degrade chromatographic performance. For simple peptide standard mixtures, 0.015% DDM was demonstrated for enabling to increase SRM signals by 3–35-fold with an average of ~20-fold improvement (Supplementary Fig. 1). We further evaluated DDM-assisted performance for single-cell level mass input of tryptic peptide mixture (i.e., 0.2 ng of acute myeloid leukemia (AML) cell lysate digests). With the addition of 0.015% DDM, the number of identified peptides (proteins) greatly increased from 63 (53) to 891(342) with ~23-fold enhancement in MS signal and a significant difference was observed between without and with DDM (Fig. 1d, e). Additional experiments from different groups have recently been conducted to further confirm the efficiency of DDM for low mass input of tryptic peptide mixture from lung cancer PC9 cell lysate digests (Supplementary Fig. 2). All these results clearly demonstrated that the feasibility of SOP-MS for analysis of sub-ng quantities of cell lysate digests (<10 mammalian cells).

We next evaluated the performance of SOP-MS by serial dilution of uniform human breast cancer MCF7 cell lysates at 0.05-2.5 ng (close to 0.5-25 cells in protein mass) in the low-bind 96-well PCR plate (Methods). For 0, 0.05, 0.25, 0.5, and 2.5 ng of proteins, after trypsin digestion the average number of identified peptides (protein groups) was 38(7), 47 (31), 214 (116), 639 (293) and 3971 (1241), respectively. With the use of a MaxQuant MBR (match-between-run) function, the number of identified peptides (protein groups) consequently increased to 110 (33), 217 (156), 928 (437), 1897 (717), and 5792 (1539), respectively (Supplementary Fig. 3a). To evaluate the quantitation accuracy of SOP-MS, we have built three types of response curves, the number of unique peptides, the number of protein groups, and the log2 extracted ion chromatogram (XIC) area as a function of low sample inputs (Supplementary Fig. 3b). All the response curves have good linearity with a correlation coefficient (R^2^) of ~0.99 from 0 to 0.5 ng, reflecting accurate quantification with a linear dynamic range for analysis of small number of cell equivalents by SOP-MS. Furthermore, SOP displayed high reproducibility with an average of Pearson correlation coefficient of ~0.90 for 0.05-0.5 ng (close to 0.5 and 5 human cells) (Supplementary Fig. 3c) and ≥0.99 between any two out of five replicates for 5 ng (Supplementary Fig. 3d). All the results have demonstrated that the ‘all-in-one’ SOP-MS enables for reproducible quantitative analysis of low mass inputs of cell lysates (close to one cell or low numbers of cells in protein mass).

### SOP-MS for label-free proteomic analysis of small tissue sections

**Fig. 2:**
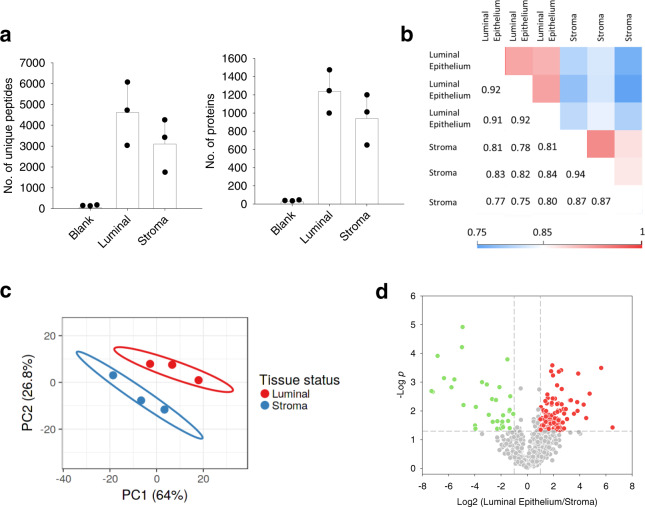
SOP-MS analysis of LCM-dissected mouse uterine tissue. **a** Number of unique peptides and protein groups identified by the MS/MS spectra only (without MBR) from three biological replicates per cell type (luminal epithelial and stroma) and three blanks. The LCM tissue size: 100 µm (length) × 100 µm (width) × 10 µm (thickness). Each LCM-dissected tissue sample is close to ~20 cells. **b** Pairwise correlation of log_10_-transformed protein LFQ intensities between any two replicates. Pearson correlation coefficients were color coded as shown on the scale at the bottom. **c** Unsupervised PCA analysis based on label-free quantification of proteins expressed in luminal epithelial and stroma cells. **d** Volcano plot of proteins differentially expressed between the two cell types from three biological replicates per cell type.

With its demonstrated improvement in analyzing low-input samples, we next evaluated whether SOP-MS can be used for label-free, global proteomics analysis of small numbers of cells derived from mouse uterine tissues (Fig. 1). Two distinct regions of luminal epithelium and stroma were dissected by laser capture microdissection (LCM) in three replicates, each with a tissue spot size of 100 µm in diameter and 10 µm in thickness (close to ~20 cells based on a recent study of small tissue sections^45^) (Supplementary Fig. 4a). These tissues were analyzed by SOP-MS for label-free proteome profiling (Fig. 1). A total of~7600 unique peptides (~1340 protein groups) were identified from luminal epithelium, and ~5200 unique peptides (~1100 protein groups) from stroma (Fig. 2a). Pairwise analysis of any two tissue samples showed Pearson correlation coefficients ranging from 0.75 to 0.94 (Fig. 2b). As expected, the correlation from the same sub-region replicates is higher than that from different sub-region replicates (Fig. 2b). This further confirmed high reproducibility of SOP-MS for processing small numbers of cells.

To evaluate whether the identified proteins can be used to specify tissue regions, we performed principal component analysis (PCA). The luminal epithelium and stroma regions were clearly segregated based on the protein expression alone with the three biological replicates from the same regions being clustered together (Fig. 2c). To identify protein features distinguishing the two regions, analysis of variance (ANOVA) was performed with a volcano plot of differentially expressed proteins (Fig. 2d), revealing ~15% of quantified proteins (~160 proteins) to be significantly different with *p* < 0.05 (Supplementary Data 2). Among the differential proteins, some of them are expected to be cell-type specific: cell junctional proteins (e.g., catenins and filamin B) and hydrolases (e.g., calpain 1 and neprilysin) for luminal epithelial cells, and extracellular matrix proteins (e.g., decorin, collagen, laminin, and fibronectin) for stromal cells (Supplementary Fig. 4b, c). Thus, SOP-MS was demonstrated to enable precise deep proteome profiling of small numbers of cells from LCM-dissected tissues.

### SOP-MS for label-free quantitative single-cell proteomics

**Fig. 3:**
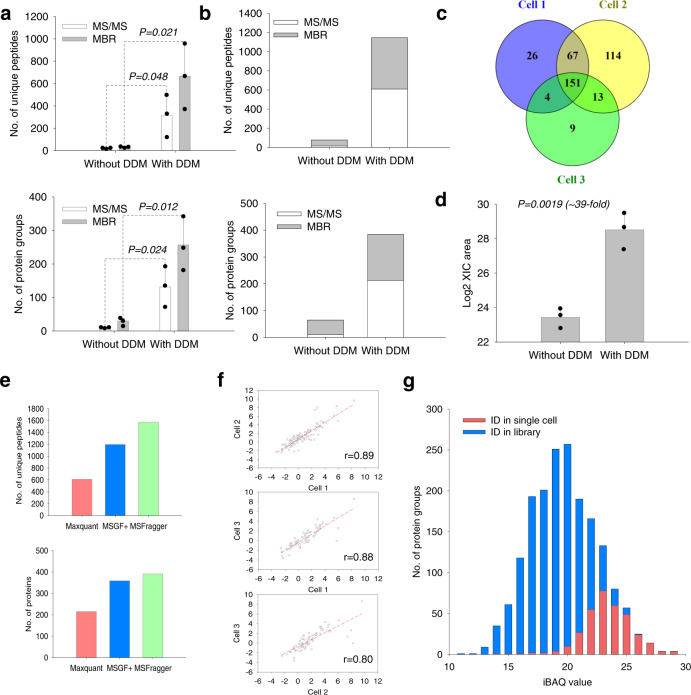
SOP-MS analysis of single MCF10A cells sorted by FACS. **a** Number of unique peptides and protein groups identified by MS/MS only and the combined MS/MS and MBR from single MCF10A cells without and with the addition of 0.015% DDM (three biological replicates per condition; *P* < 0.05 between without and with DDM). **b** Total number of unique peptides and protein groups identified by MS/MS only and the combined MS/MS and MBR across all three biological replicates without and with the addition of 0.015% DDM. **c** Venn diagram showing the number of protein groups identified from each of three single MCF10A cells with the addition of 0.015% DDM by the combined MS/MS and MBR. **d** The summed total XIC peak area for all quantifiable peptides from single MCF10A cells (three biological replicates per condition; *P* < 0.05 between without and with DDM). **e** Total number of unique peptides and protein groups identified by the MS/MS spectra alone from all three biological replicates using three common search tools (MaxQuant, MSGF+ , and MSFragger). **f** Pairwise correlation of protein LFQ intensities between any two replicates with the Pearson correlation coefficient. **g** Distribution of protein abundance for all proteins identified from single MCF10A cells and 10 ng MCF10A cell lysate digests. Library was built with 10 ng MCF10A cell lysate digests.

With the demonstrated performance for small numbers of cells, we evaluated whether SOP-MS can be used for proteomic analysis of single mammalian cells. Single cells were sorted directly into single low-bind PCR tubes (one cell per tube) by fluorescence-activated cell sorting (FACS). Single MCF10A cells were processed without and with 0.015% DDM (three biological replicates per condition) in parallel by SOP followed by LC-MS analysis (Fig. 1). With the DDM additive, the average number of unique peptides identified from biological triplicates was 313, resulting in the identification of 131 protein groups with the MS/MS spectra alone (i.e., without MBR) (Fig. 3a). By contrast, without DDM the average number of unique peptides was only 6, corresponding to 5 protein groups. Furthermore, a significant difference was observed between without and with DDM (Fig. 3a). This result strongly suggests that without the DDM additive the ‘all-in-one’ one-pot method cannot effectively process single cells for proteomic analysis, consistent with our observation for cell lysate digests and peptide standards.

To increase the number of identified unique peptides (protein groups), other commonly used proteomic algorithms were used to reanalyze the single-cell data. With the use of MBR function in MaxQuant, the average protein identifications were increased to 229, and a total of 384 protein groups were identified across three biological replicates for single MCF10A cells (Fig. 3b). 151 protein groups were commonly identified for all 3 single MCF10A cells, and an average of ~53% protein groups overlapped between any two single MCF10A cells, suggesting cell-to-cell variability (Fig. 3c). An average of ~39-fold enhancement in MS signal was observed with a significant difference between samples without and with DDM (Fig. 3d), which further confirmed the importance of using DDM additive for single-cell processing. When compared to MaxQuant search with identification of a total 215 protein groups by the MS/MS spectra alone across three MCF10A biological replicates, other two common software tools MSGF+ and MSFragger were evaluated with enabling identification of 359 protein groups for MSGF + and 391 protein groups for MSFragger (Fig. 3e). These results have further confirmed that SOP-MS enables the confident detection of hundreds of proteins from single human cells. Among the three software tools, MaxQaunt is the most commonly used tool for label-free quantification. Unless otherwise mentioned, MaxQuant was used for quantitative analysis of all the single-cell proteomics data. We next evaluated the reproducibility of SOP-MS for quantitative single-cell proteomic analysis. High reproducibility was demonstrated with Pearson correlation between any two single cells of 0.80-0.89 for single MCF10A cells (Fig. 3f). To evaluate the measurement reliability by SOP-MS, we compared the abundance distribution of proteins identified in single cells with that from 10 ng MCF10A cell lysate digests. As expected, most proteins identified in single cells were highly abundant and above the median abundance of the 10 ng MCF10A cell lysate digests (Fig. 3g). Therefore, SOP-MS enables precise, quantitative, label-free single-cell proteomics.

**Fig. 4:**
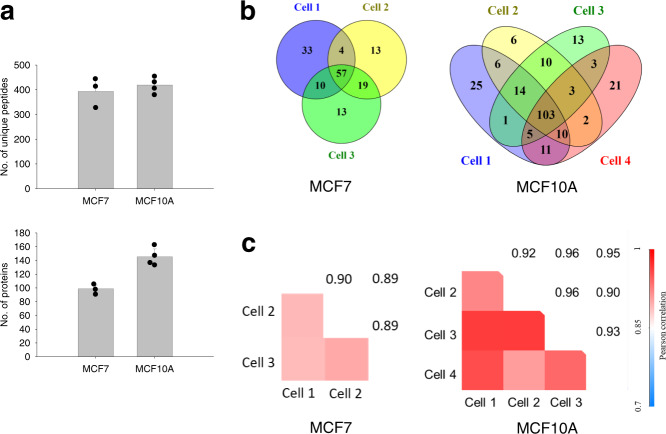
Validation of SOP-MS for quantitative single-cell proteomics analysis. **a** Number of unique peptides and protein groups identified by the MS/MS spectra alone from three single MCF7 cells and four single MCF10A cells (from newly cultured MCF10A cells) sorted by FACS. **b** Venn diagram showing the number of protein groups identified from each of single MCF7 or MCF10A cells. **c** Pairwise correlation of log_10_-transformed protein LFQ intensities between any two replicates from the three single MCF7 cells and the 4 single MCF10A cells. Pearson correlation coefficients were color coded as shown on the scale at the bottom. Half of the sample injection was used for the analysis of single MCF7 cells (i.e., 0.5 single MCF7 cells for MS analysis).

To validate SOP-MS for single-cell proteomics analysis we performed an independent experiment for 4 single cells sorted by FACS from newly cultured MCF10A cells. An average of 146 protein groups were identified with the MS/MS spectra (Fig. 4a) and 103 protein groups were commonly identified for all the 4 single MCF10A cells (Fig. 4b). An average of ~64% protein groups overlapped between any two single cells, suggesting lower cell-to-cell variability when compared to the above 3 single MCF10A cells (Fig. 3c). This was further confirmed by the higher median correlation coefficient (~0.94) (Fig. 4c) than that from the above 3 single MCF10A cells (Fig. 3f). In addition, SOP-MS was used for the analysis of different types of cells, 3 single MCF7 cancer cells with half of the sample injection (i.e., ~0.5 single cells for MS analysis) to mimic other small-size single mammalian cells. An average of 98 protein groups were identified from half of the single MCF7 cells with a correlation coefficient of 0.9 (Fig. 4). All these results further confirmed the high reproducibility of SOP-MS for reliable label-free quantification of 100s of proteins from single mammalian cells.

### Application of SOP-MS to single cells derived from a PCDX model

**Fig. 5:**
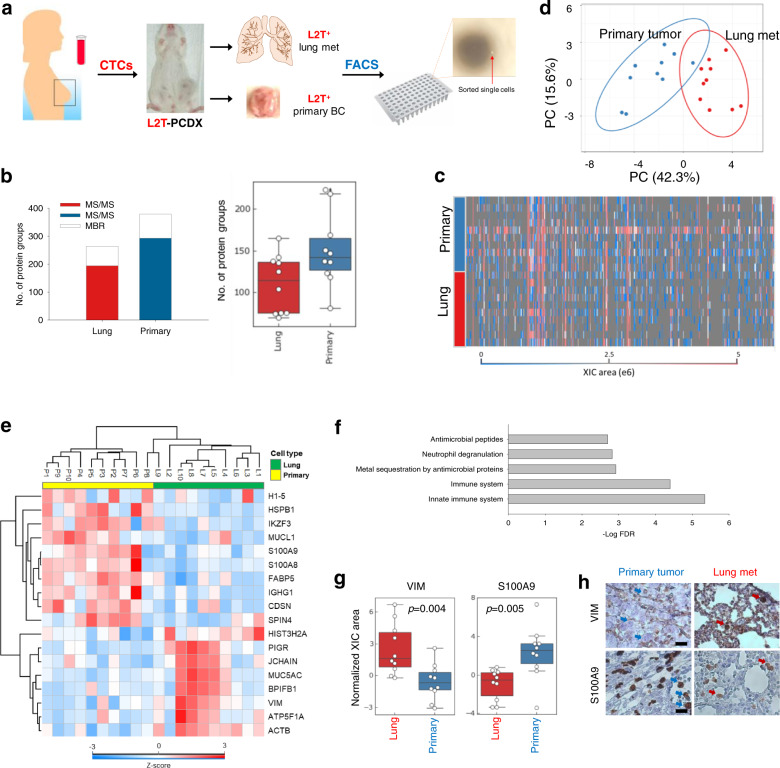
SOP-MS analysis of single cells derived from a PCDX model. **a** Schematic workflow of SOP-MS analysis of single cells derived from a PCDX model. CTCs from a breast cancer patient (NU-205) were isolated and implanted into NSG mouse mammary fat pads to generate the PCDX-205 mouse. The PCDX was verified (Supplementary Fig. 5) and transduced to express Luc2-tdTomato (L2T). Labeled primary PCDX and lungs were harvested for tissue dissociation and single-cell sorting. L2T^+^ single cells from the primary tumor and lung metastases were collected individually into single well of a 96-well PRC plate for SOP-MS analysis. **b** Total number of unique peptides and protein groups identified by the combined MS/MS and MBR across all 10 single lung metastatic cells or 10 single primary tumor cells, and the number of protein groups identified by the combined MS/MS and MBR for each single lung metastatic cells or single primary tumor cells. **c** Heatmap showing the total XIC peak area for each protein group identified by the MaxQuant MBR from either the 10 single primary tumor cells or the 10 single lung metastatic cells. **d** PCA analysis based on label-free quantification of proteins expressed in single cells from primary tumor and lung metastasis (10 single cells for each cell type). **e** Heatmap showing 18 differentially expressed proteins between single cells from primary tumor and lung metastasis. **f** Bar chart for pathway annotation. The bars represent the annotated pathways within proteins significantly expressed between two types of single cells. **g** Box plots showing the normalized expression levels of VIM (left) and S100A9 (right) between single lung metastatic cells and single primary tumor cells by using SOP-MS (10 single cells per cell type). **h** Immunohistochemistry (IHC) images of primary tumors and lung metastases, stained for VIM (top) and S100A9 (bottom). Arrows indicate representative, positive staining tumor cells. Scale bar = 50 µm.

To demonstrate the potential applications of SOP-MS to cancer research as well as to evaluate whether the identification of hundreds of relatively abundant proteins can provide meaningful biological insights into cellular heterogeneity, we applied SOP-MS for single-cell proteomic analysis of primary tumors and early lung metastases in a PCDX mouse model generated from patient CTCs (Supplementary Fig. 5). After dissociation of luciferase 2-tdTomato (L2T)-labeled PCDX tissues, single L2T^+^ tumor cells were sorted by FACS into 96-well PCR plates (one cell per well) with ten from propagated CTCs (primary) and ten from metastases (lung) (Fig. 5a). With the MaxQuant MBR function, a total of 265 proteins were identified across all 10 single lung metastatic cells with the range of 69-163 protein groups for each single cells, and a total of 379 proteins identified across all 10 single primary tumor cells with the range of 81–223 protein groups for each single cells (Fig. 5b). The total XIC peak area for each protein group across the 20 single cells was presented as a heatmap for an overview of protein group detection (Fig. 5c). The higher number of protein identification from single primary tumor cells is consistent with their relatively larger size when compared to lung cells (breast tumor cells: ~12 µm in diameter^46^ and lung cells: ~8 µm in diameter^47^), reflecting the reliability of SOP-MS for single-cell proteomic analysis.

Unsupervised PCA analysis has shown distinct clustering of proteins from the primary CTCs versus the lung metastases (Fig. 5d), with significant abundance changes for 18 proteins between the two cell types (Fig. 5e and Supplementary Data 3). Cellular heterogeneity within the same cell type and between the two different cell types was clearly observed based on protein abundance achieved by label-free quantification (Fig. 5d). Based on pathway analysis, many of these proteins differentially expressed in the early metastases are annotated as immune-related proteins (e.g., S100 calcium-binding family proteins A8 and A9, IGHG1, PIGR, and BPIFB1) (Fig. 5f). This may infer tumor cell alterations enabling immune evasion in response to the dynamic selection pressure of anti-tumor immunity from the transition of primary tumor cells to early metastasis. With literature mining, many proteins showing a reduced abundance in the lung metastases are associated with epithelial cell differentiation (e.g., CDSN) or epithelial cancers (e.g., S100A family proteins^48^ and MUCL1 small breast epithelial mucin^49^), consistent with the cell-type plasticity between primary tumor and early metastasis. Notably, in the lung metastases the EMT markers, vimentin (VIM), MU5AC^50^ and PIGR^51^, displayed significant upregulation (Fig. 5e), suggesting the occurrence of EMT in early micrometastatic cells. Meanwhile, downregulated two chaperone proteins (HSPB1^52,53^ and FABP5^54^) reported to promote EMT, may infer altered adaptation states in the lung metastatic cells (Fig. 5e).

To further validate label-free MS quantification, two representative proteins, VIM and S100A9, were selected with median expression upregulated and downregulated by 4.7 and 8.6 in the lung metastatic cells, respectively (Fig. 5g). The two proteins were measured with immunohistochemistry (IHC) staining of the primary tumor and lung tissue sections from the original PCDX model used for sorting single L2T^+^ tumor cells. Results from IHC staining are in agreement with the data from label-free MS quantification (Fig. 5h), which confirmed reliable single-cell proteomic quantification with SOP-MS.

## Discussion

SOP-MS is a convenient robust method for label-free single-cell proteomics, where single cells are processed in either low-bind single tubes or multi-well plates which are routinely used for single-cell genomics and transcriptomics. The performance of SOP-MS (e.g., sensitivity, reproducibility, and quantitation accuracy) was demonstrated by label-free MS analysis of low mass inputs from a serial dilution of uniform MCF7 cell lysates, LCM-dissected small tissue sections, and FACS-sorted single cells. Based on the actual MS/MS spectra for reliable protein identification (without using the MBR function) which is the cornerstone of MS-based proteomics, SOP-MS can identify ~146 protein groups from single human cells, higher than ~128 for iPAD1-MS^24^ and 51 for OAD-MS^25^ and ~1.4-2.5-fold lower than ~211-362 for nanoPOTS-MS^55–57^ (Supplementary Table 1), and ~1200 proteins from small tissue sections (close to ~20 cells). Comparative analysis of single MCF10A cells using both SOP-MS and nanoPOTS-MS has shown that the number of protein groups from SOP-MS is ~1.6-fold lower than that from nanoPOTS-MS and ~60% of protein groups from SOP-MS overlapped with the protein groups from nanoPOTS-MS (Supplementary Fig. 6 and Supplementary Table 1). Most importantly, unlike all currently available label-free single-cell proteomics methods that need specific devices and are difficult to access by research community, SOP-MS has advantages in terms of high compatibility with cell sorting or tissue collection systems and LC-MS analysis using single tubes or multi-well plates (Fig. 1a–c), and high flexible scalability shifting from single tube to multi-well plate for one-pot sample preparation. Thus, SOP-MS is easy to be widely adopted by research community for broad applications. Furthermore, automation of the whole ‘all-in-one’ sample preparation workflow can be readily achieved for high sample throughput by using commercially available liquid handlers for precisely dispensing µL or sub-µL reagent solution. Therefore, SOP-MS represents a breakthrough in technology for label-free MS-based single-cell proteomics.

With its demonstration for label-free MS analysis, SOP-MS can be equally used for other types of single-cell proteomic analysis (e.g., targeted proteomics and TMT-based MS analysis). It can also be used for the analysis of other ultrasmall precious clinical specimens (e.g., rare CTCs and tissues from fine-needle aspiration biopsy). We have initially evaluated the integration of our recently developed TMT-based BASIL strategy^58^ into SOP-MS for multiplexed analysis of 9 single MCF10A cells. A median correlation coefficient of ~0.95 was achieved (Supplementary Fig. 7d) primarily due to high recovery and reproducibility of SOP-MS.

Future developments will focus on improvements in detection sensitivity and sample throughput for rapid deep proteome profiling of single mammalian cells. Enhancing detection sensitivity could be achieved by effective integration of ultralow-flow LC or capillary electrophoresis (CE) and a high-efficiency ion source/ion transmission interface with the most advanced MS platform. Further improvement can be gained by further reducing sample loss (e.g., systematic evaluation of different types of MS-friendly surfactants) and increasing reaction kinetics through reducing processing volume from 10–15 µL down to 1–2 µL with automated small-volume liquid handling (e.g., automated MANTIS liquid handler). All these improvements in detection sensitivity will lead to greatly increase the measurement reliability (e.g., more high-quality MS/MS spectra) as well as the number of identified peptides/protein groups. Sample throughput could be increased by using ultrafast high-resolution ion mobility-based gas-phase separation (e.g., SLIM^59^) to replace current slow liquid-phase (LC or CE) separation, and effective integration of liquid- and gas-phase separations (e.g., SLIM^59^ or FAIMS^60^) for greatly reducing separation time but without trading off separation resolution. Alternatively, sample multiplexing with isobaric barcoding and implementation of a multiple LC column system can also be considered to increase sample throughput. All these improvements could lead to a more powerful SOP-MS platform and will certainly close the gap between single-cell proteomics and single-cell transcriptomics or genomics.

When compared to proteomic analysis of bulk cells that only provides the averaged expression signal, single-cell proteomics can provide a clean signal for single cells of interest without signal contribution from other types of cells, allowing to uncover new biological discoveries. When applied for the analysis of single cells derived from a clinically relevant PCDX model, SOP-MS can reveal distinct protein signatures between primary and metastatic tumors as well as cellular heterogeneity within the same cell type. Proteins with altered expression levels are involved in tumor immunity (e.g., S100A family members^61^), epithelial cell differentiation (e.g., CDSN), and EMT (vimentin^38,62^), suggesting possible selective pressure for immune evasion and cell state plasticity. The data provide a clear path for future mechanistic studies of cancer metastasis with the potential to guide targeted cancer therapy. SOP-MS analysis of single cells is underway to reveal robust protein signatures related to physiological and pathological states at the single-cell resolution. Furthermore, with its demonstration for analysis of CTC-derived single cells, SOP-MS can be equally applied to clinically important patient CTCs that link disseminated and primary tumors. Thus, it has great potential for liquid biopsy-guided diagnostic and prognostic applications as well as for rational therapeutic intervention.

In summary, we report an easily implementable SOP-MS method that capitalizes on using surfactant-assisted one-pot sample preparation to reduce the surface adsorption losses for label-free single-cell proteomics. Label-free quantitative proteome profiling of single cells can be achieved with easily accessible sample preparation devices (single tubes or multi-well plates) and standard LC-MS platforms. With its convenient features, SOP-MS can be readily implemented in any MS laboratory for single-cell proteomic analysis. The application of SOP-MS to single cells derived from a PCDX model demonstrated its power for precise characterization of cellular heterogeneity and discovery of distinct protein signatures related to breast cancer metastasis. With improvements in detection sensitivity and sample throughput as well as automation for high sample throughput, we believe that SOP-MS has great potential to close the gap between single-cell proteomics and single-cell transcriptomics, and could open an avenue for single-cell proteomics with broad applicability in the biological and biomedical research.

## Methods

### Human sample collection and animal studies

The human blood analyses for breast cancer patients were approved by the Institutional Review Boards at Northwestern University and complied with NIH guidelines for human subject studies. Animal procedures and experimental procedures have been performed under approval by Northwestern University Animal Care and Use Committee (ACUC) and complied with the NIH Guidelines for the Care and Use of Laboratory Animals. 8-10 weeks old female NSG mice were used for implantation of human breast cancer PCDX models and kept in specific pathogen-free facilities in the Animal Resources Center at Northwestern University. Breast tumors were harvested after 2-3 months and confirmed as a human PCDX with positive expression of human epithelial markers EpCAM, HER2, and CD44 as well as negative expression of mouse H-2Kd.

### Reagents

n-Dodecyl β-D-maltoside (DDM), dithiothreitol (DTT), iodoacetamide (IAA), ammonium bicarbonate, acetonitrile, and formic acid were obtained from Sigma-Aldrich (St. Louis, MO). Promega trypsin gold was purchased from Promega Corporation (Madison, WI). Synthetic heavy peptides labeled with ^13^C/^15^N on the C-terminal arginine or lysine were purchased from New England Peptide (Gardner, MA).

### Cell culture

The MCF10A (MCF7) breast cancer cell line was obtained from the American Type Culture Collection (Manassas, VA) and was grown in culture media^63^. Briefly, MCF10A (MCF7) cells were cultured and maintained in 15 cm dishes in ATCC-formulated Eagle’s minimum essential medium (Thermo Fisher Scientific) supplemented with 0.01 mg/mL human recombinant insulin and a final concentration of 10% fetal bovine serum (Thermo Fisher Scientific, Waltham, MA) with 1% penicillin/streptomycin (Thermo Fisher Scientific). Cells were grown at 37 °C in 95% O_2_ and 5% CO_2_. Cells were seeded and grown until near confluence.

### MCF7 cell lysates

MCF7 cells were rinsed twice with ice-cold phosphate-buffered saline (PBS) and harvested in 1 mL of ice-cold PBS containing 1% phosphatase inhibitor cocktail (Pierce, Rockford, IL) and 10 mM NaF (Sigma-Aldrich). Cells were centrifuged at 1500 rpm for 10 min at 4 °C, and excess PBS was carefully aspirated from the cell pellet. Cell pellets were resuspended in ice-cold cell lysis buffer (250 mM HEPES, 8 M urea, 150 mM NaCl, 1% Triton X-100, pH 6.0) at a ratio of ~3:1 lysis buffer to cell pellet. Cell lysates were centrifuged at 14,000 rpm at 4 °C for 10 min, and the soluble protein fraction was retained. Protein concentrations were determined by the BCA assay (Pierce).

### Fluorescence-assisted cell sorting (FACS) of single cells

Prior to cell collection, PCR tubes or 96-well PCR plates were pretreated with 0.1% DDM for coating the surface and later the DDM solution was removed. The pretreated PCR tubes or 96-well PCR plates were air-dried in the fume hood. To avoid cell clumping, after detaching they were dispersed into a single-cell suspension by passing three times through a 25-gauge needle. The cells were suspended in PBS, and pelleted by centrifuging 5 min at 500 *g*. This process was repeated five times to remove the remaining PBS and trypsin. After that the cells were resuspended in PBS and passed through a 35 μm mesh cap (BD Biosciences, Canaan, CT) to remove large aggregates. A BD Influx flow cytometer (BD Biosciences, San Jose, CA) was used to deposit cells into the precoated PCR tubes. Alignment into a Hard-Shell 96-well PCR plate (Bio-Rad, Hercules, CA) was done using fluorescent beads (Spherotech, Lake Forest, IL), after which the coated PCR tubes were placed into the plates for cell collection. For unstained MCF10A cells, forward and side scatter detectors were used for cell identification. Once sorting gates were established, cells were sorted into the PCR tubes using the 1-drop single sort mode. After isolation of the desired number of cells into the PCR tube, the isolated cells were immediately centrifuged at 1000 *g* for 10 min at 4 °C to keep the cells at the bottom of the tube to avoid potential cell loss. The PCR tubes with the isolated cells were stored in a −80 °C freezer until further analysis.

### Laser capture microdissection (LCM) of tissue sections

Prior to LCM experiments, a cap of PCR tube was prepopulated with a 5 µL water droplet. Laser capture microdissection (LCM) was performed on a PALM MicroBeam system (Carl Zeiss MicroImaging, Munich, Germany). Voxelation of the tissue section was achieved by selecting the area on the tissue using PalmRobo software, followed by tissue cutting and catapulting. Mouse uterine tissues containing two distinct cell types (luminal epithelium and stroma) were cut at an energy level of 42 and with an iteration cycle of 2 to completely separate 100 µm × 100 µm tissue voxels at a thickness of 10 µm. The “CenterRoboLPC” function with an energy level of delta 10 and a focus level of delta 5 was used to catapult tissue voxels into the cap. The “CapCheck” function was activated to confirm successful sample collection from tissue sections to water droplets. After tissue collection into the droplet of the cap, the PCR tube was immediately centrifuged at 1000 g for 10 min at 4 °C to keep collected tissues at the bottom of the tube to avoid potential sample loss. The collected samples were processed directly or stored at −80 °C until use.

### PCDX model generation and dissociation of PCDX tumors and lungs

The PCDX-205 model was created by implanting prospective CTCs upon lysis of red blood cells (lysis buffer Sigma cat# R7757) and depletion of CD45^+^ PBMCs (Miltenyi Biotec Depletion column cat#130-042-901) from the blood cells of a breast cancer patient (NU-205) into the mammary fat pads of NSG mice. Breast tumors were harvested after 2-3 months and confirmed as a human PCDX with positive expression of human epithelial markers EpCAM, HER2, and CD44 as well as negative expression of mouse H2K^d^. Tumor cells were lentiviral labeled by L2T^64^ which was generated by using the Luc2 and td Tomato sequences with connection by the short linker, 5′-GGAGATCTAGGAGGTGGAGGTA-GCGGTGGAGGTGGAAGCCAGGATCC-3′. The L2T gene sequence was removed from a pCDNA3.1^+^ vector and placed within the pFUG lentiviral vector using traditional blunt-end cloning. The spontaneous lung metastases were detected by IVIS of the lungs when dissected from the mice.

L2T^+^ PCDX-205 primary tumors and the lungs were harvested and briefly washed in PBS. Tissue was transferred to a Petri dish containing 10 mL dissociation media (RPMI 1640 media with 20 mM HEPES buffer), then minced into fine pieces. 400 µL of Liberase TH enzyme (Roche cat# 5401135001) and 100 Units of DNase enzyme (Sigma cat# D4263) were added to the dissociation media, and the Petri dishes containing the tissues were transferred to an incubator at 37 °C and 5% CO_2_ for 2 h to complete dissociation. Tissue suspension was mixed every 15 min using a 10 mL serological pipette to aid dissociation. After tissue was completely digested into single cells, the solution was transferred to a 50 mL conical tube. The original petri dish was washed with 15 mL RPMI media containing 2% fetal bovine serum (FBS) (Sigma) and 1% penicillin/streptomycin (Gibco) and the contents transferred to a 50 mL conical tube containing the tissue solution to stop the dissociation reaction. Samples were centrifuged at 300 *g* for 5 min at 4 °C, and the supernatant was removed. Samples were resuspended in 4 mL Red Blood Cell Lysing Buffer (Sigma) and kept on ice for 10 min, after which 20 mL of HBSS (Corning) was added to samples and centrifuged at 300 *g* for 5 min at 4 °C and the supernatant was removed. Samples were resuspended in 20 mL HBSS and filtered with a 40 µm filter. Cell numbers were counted, and samples were stored on ice until ready for use.

### Single-cell sorting of patient CTCs from PCDXs and early metastases to the lungs

Cells from dissociated tumor and lung tissues were washed in PBS and then centrifuged at 300*g* for 5 min at 4 °C. Samples were resuspended in 2% FBS in PBS. MDA-MB-231 cells were collected and suspended in 2% FBS in PBS to serve as a tdTomato (L2T)-negative control for flow analysis. Cancer cells from the tumor and lung samples were sorted based on L2T expression. L2T^+^ tumor cells of the lung metastases were initially sorted into 10% FBS in PBS prior to single-cell sorting, and each of the L2T^+^ single cells from the primary tumor and lung metastases was sorted into 5 µL H_2_O in a single tube of a 96-tube PCR plate. Plates were sealed, briefly spun on a microplate centrifuge, and stored at −80 °C until later SOP-MS analysis.

### Immunohistochemistry staining

Formalin-fixed and paraffin-embedded tissues were processed and sectioned according to routine protocols. Heat mediated antigen retrieval was used prior to all staining procedures. Tissues were incubated with vimentin antibody (1:200 dilution, clone D21H3, Cell Signaling Technology) or S100A9 antibody (1:100 dilution, provided by Dr. Philippe Tessier at Laval University) overnight at 4 °C. Antigen was detected using the EnVision+ Dual Link System (Dako) and counterstained with hematoxylin. Images were taken using a Leica DM4000B microscope and a Leica MC120 HD camera with a 40× objective.

### Cell lysis, reduction, alkylation, and trypsin digestion

For FACS-isolated cells, 2 µL of 0.1% DDM in 25 mM ammonium bicarbonate (ABC) was added to the PCR tube or each well of the 96-well plate. Intact cells were sonicated at 1-min intervals for 5 times over ice for cell lysis and centrifuged for 3 min at 3000 g. 0.3 µL of 100 mM DTT in 25 mM ABC was added to the PCR tube. Samples were incubated at 75 °C for 1 h for denaturation and reduction. After that, 0.5 µL of 60 mM IAA in 25 mM ABC was added to the PCR tube. Samples were incubated in the dark at room temperature for 30 min for alkylation. The reduction and alkylation steps appear optional: there is no apparent difference in protein identification and quantification between samples with and without reduction and alkylation. 2 µL of 1 ng/µL trypsin (Promega) in 25 mM ABC was added to the PCR tube or the 96-well plate at a total amount of 2 ng. Samples were digested for ~3–4 h at 37 °C with gentle sharking at ~500 g. After digestion, 0.5 µL of 5% FA was added to the tube to stop enzyme reaction. The final sample volume was adjusted to ~10–15 μL with the addition of 25 mM ammonium bicarbonate (triethylammonium bicarbonate for TMT samples) for direct LC injection. The sample PCR tube was inserted into the LC vial or the 96-well PCR plate was sealed with a matt. They were either analyzed directly or stored at −20 °C for later LC-MS analysis. For the integrated SOP-BASIL-MS analysis, the digested peptides from single MCF10A cells were labeled with different TMT reagents as sample channels, and 10 ng of peptides from bulk MCF10A cell digests were labeled with TMT126 as the carrier channel. The TMT126 labeled carrier channel peptides were equally distributed to each sample channel, and all the samples were combined together to form one single sample. The combined channel sample was desalted by using a simple reversed phase-based Stage Tip^65^.

For LCM-dissected tissue sections, 1.5 µL of cell lysis buffer containing 0.2% DDM and 5 mM DTT was added to the PCR tube and incubated at 80 °C for 60 min for cell lysis and protein denaturation. IAA was added to the PCR tube with the final concentration of 10 mM. Samples were incubated in the dark at room temperature for 30 min. After that they were diluted by the addition of 25 mM ammonium bicarbonate to reduce the DDM concentration to 0.02%. The mixed Lys-C and trypsin were added to the PCR tube with the final enzyme concentration of 0.5 ng/µL (i.e., a total of 5 ng for the final processing volume of 15 μL). The sample was gently mixed at 850 rpm for 3 min, and then incubated at 37 °C overnight (∼16 h) for digestion. After digestion, 1 µL of 5% FA was added to the PCR tube to stop enzyme reaction. The sample PCR tube was inserted into the LC vial and the sample was either directly analyzed or stored at −20 °C for later LC-MS analysis.

### LC-MS/MS analysis

The single-cell digests were analyzed using a commonly available Q Exactive Plus Orbitrap MS (Thermo Scientific, San Jose, CA). The standard LC system consisted of a PAL autosampler (CTC ANALYTICS AG, Zwingen, Switzerland), two Cheminert six-port injection valves (Valco Instruments, Houston, USA), a binary nanoUPLC pump (Dionex UltiMate NCP-3200RS, Thermo Scientific), and an HPLC sample loading pump (1200 Series, Agilent, Santa Clara, USA). Both SPE precolumn (150 µm i.d., 4 cm length) and LC column (50 µm i.d., 70-cm Self-Pack PicoFrit column, New Objective, Woburn, USA) were slurry-packed with 3-µm C18 packing material (300-Å pore size) (Phenomenex, Terrence, USA). Sample was fully injected into a 20 µL loop and loaded onto the SPE column using Buffer A (0.1% formic acid in water) at a flow rate of 5 µL/min for 20 min. The concentrated sample was separated at a flow rate of 150 nL/min and a 75 min gradient of 8-35% Buffer B (0.1% formic acid in acetonitrile). The LC column was washed using 80% Buffer B for 10 min and equilibrated using 2% Buffer B for 20 min. Q Exactive Plus Orbitrap MS (Thermo Scientific) was used to analyze the separated peptides. A 2.2 kV high voltage was applied at the ionization source to generate electrospray and ionize peptides. The ion transfer capillary was heated to 250 °C to desolvate droplets. The data-dependent acquisition mode was employed to automatically trigger the precursor scan and the MS/MS scans. Precursors were scanned at a resolution of 35,000, an AGC target of 3 × 10^6^, a maximum ion trap time of 50 ms (100 ms for CTC single-cell analysis). Top-10 precursors were isolated with an isolation window of 2, an AGC target of 2 × 10^5^, a maximum ion injection time of 300 ms (for CTC single-cell analysis, the AGC target of 2 × 10^5^ and 500 ms ion injection time was used), and fragmented by high energy collision with an energy level of 32%. A dynamic exclusion of 30 s was used to minimize repeated sequencing. MS/MS spectra were scanned at a resolution of 17,500.

### Data analysis

The freely available open-source MaxQuant software was used for protein identification and quantification. The MS raw files were processed with MaxQuant (Version 1.5.1.11)^66,67^ and MS/MS spectra were searched by Andromeda search engine against the against a human (or mouse) UniProt database (fasta file dated April 12, 2017) (with the following parameters: tryptic peptides with 0-2 missed cleavage sites; 10 ppm of parent ion tolerance; 0.6 Da of fragment ion mass tolerance; variable modifications (methionine oxidation). Search results were processed with MaxQuant and filtered with a false discovery rate ≤1%. When a peptide library was available, the match between runs (MBR) function was selected to increase proteome coverage. Protein quantification was performed by using the label-free quantitation (LFQ) function. Contaminants were removed from the peptides.txt file prior to use for downstream statistical analysis. Biological functions and signaling pathways were analyzed by using DAVID Bioinformatics Resources (Version 6.8)^68^ and Peruses (Version 1.6.2.1)^69^, and protein-protein association network analysis was performed by the latest version of STRING (Version 11.0)^70^.

### Statistics and reproducibility

At least three biological or technical replicates were used to evaluate reproducibility for sample recovery and SOP-MS. No data exclusion was performed, and no randomization or blinding methods were used in data analysis. After label-free quantification with MaxQuant MBR, the extracted ion chromatogram (XIC) areas of the identified protein groups were log2 transformed, and then normalized by the median value of each column. The proteins containing at least 50% valid values in one group were kept in the data matrix, and the missing values were imputed by the normal distribution in each column with a width of 0.3 and a downshift of 1.8 by using Perseus (Version 1.6.2.1)^69^. The non-supervised PCA analysis was used to generate PCA plot. We further used Anova *t* test to prioritize significantly differentiated proteins (*p* < 0.05, FDR < 0.2) for the heatmap generation. The extracted data were further processed and visualized with Microsoft Excel 2017.

### Reporting summary

Further information on research design is available in the Nature Research Reporting Summary linked to this article.

## Supplementary information

Supplementary Information

Description of Additional Supplementary Files

Reporting Summary

Supplementary Data 1

Supplementary Data 2

Supplementary Data 3

Supplementary Data 4

## Data Availability

The RAW global MS data and the identified protein groups from MaxQuant have been deposited in Japan ProteOme STandard Repository (jPOST: https://repository.jpostdb.org/)^71^. The accession codes: JPST000866 for jPOST and PXD019626 for ProteomeXchange. The Skyline-processed SRM results and the RAW targeted MS data for Supplementary Data 1 can be accessed without restrictions at Panorama (Access link: https://panoramaweb.org/AMPFxF.url) and ProteomeXchange (Accession code: PXD022827), respectively. The index of all the source data for Figs. 1–5 was listed in Supplementary Table 4.
