# Supplementary material for: Surfactant-assisted one-pot sample preparation for label-free single-cell proteomics
Source: Commun Biol. 2021 Mar 1;4:265. doi: 10.1038/s42003-021-01797-9 (PMC7921383; doi:10.1038/s42003-021-01797-9)
Supplement: Supplementary file 1 — Supplementary Information [file 42003_2021_1797_MOESM1_ESM.pdf]

Supplementary Information

Surfactant-assisted one-pot sample preparation for label-free single-cell proteomics

Chia-Feng Tsai[1#], Pengfei Zhang[1,2#], David Scholten[3#], Kendall Martin[1], Yi-Ting Wang[1], Rui Zhao[4], William B. Chrisler[1], Dhwani B. Patel[3], Maowei Dou[4], Yuzhi Jia[3], Carolina Reduzzi[3], Xia Liu[3], Ronald J. Moore[1], Kristin E. Burnum-Johnson[1], Miao-Hsia Lin[5], Chuan-Chih Hsu[6], Jon M. Jacobs[1], Jacob Kagan[7], Sudhir Srivastava[7], Karin D. Rodland[1], H. Steven Wiley[4], Wei-Jun Qian[1], Richard D. Smith[1], Ying Zhu[4], Massimo Cristofanilli[8,9], Tao Liu[1¶], Huiping Liu[3,8,9¶], Tujin Shi[1¶]

[1]Biological Sciences Division, Pacific Northwest National Laboratory, Richland, Washington 99354, USA
[2]NHC Key Laboratory of Cancer Proteomics, Department of Oncology, Xiangya Hospital, Central South University, Changsha, Hunan, 410008, P.R. China
[3]Department of Pharmacology, Feinberg School of Medicine, Northwestern University, Chicago, Illinois 60611, USA.
[4]Environmental Molecular Sciences Laboratory, Pacific Northwest National Laboratory, Richland, Washington 99354, USA
[5]Graduate Institute of Microbiology, College of Medicine, National Taiwan University, Taipei 10617, Taiwan
[6]Institute of Plant and Microbial Biology, Academia Sinica, Taipei 11529, Taiwan
[7]Cancer Biomarkers Research Group, Division of Cancer Prevention, National Cancer Institute, Bethesda, Maryland 20892, USA
[8]Division of Hematology and Oncology, Department of Medicine, Feinberg School of Medicine, Northwestern University, Chicago, Illinois 60611, USA.
[9]Robert H. Lurie Comprehensive Cancer Center, Feinberg School of Medicine, Northwestern University, Chicago, Illinois 60611, USA.

[¶]Corresponding authors:
Dr. Tujin Shi
Pacific Northwest National Laboratory
Richland, WA 99352
Email: tujin.shi@pnnl.gov

Dr. Huiping Liu
Northwestern University
Chicago, IL 60611
Email: huiping.liu@northwestern.edu

Dr. Tao Liu
Pacific Northwest National Laboratory
Richland, WA 99352
Email: tao.liu@pnnl.gov

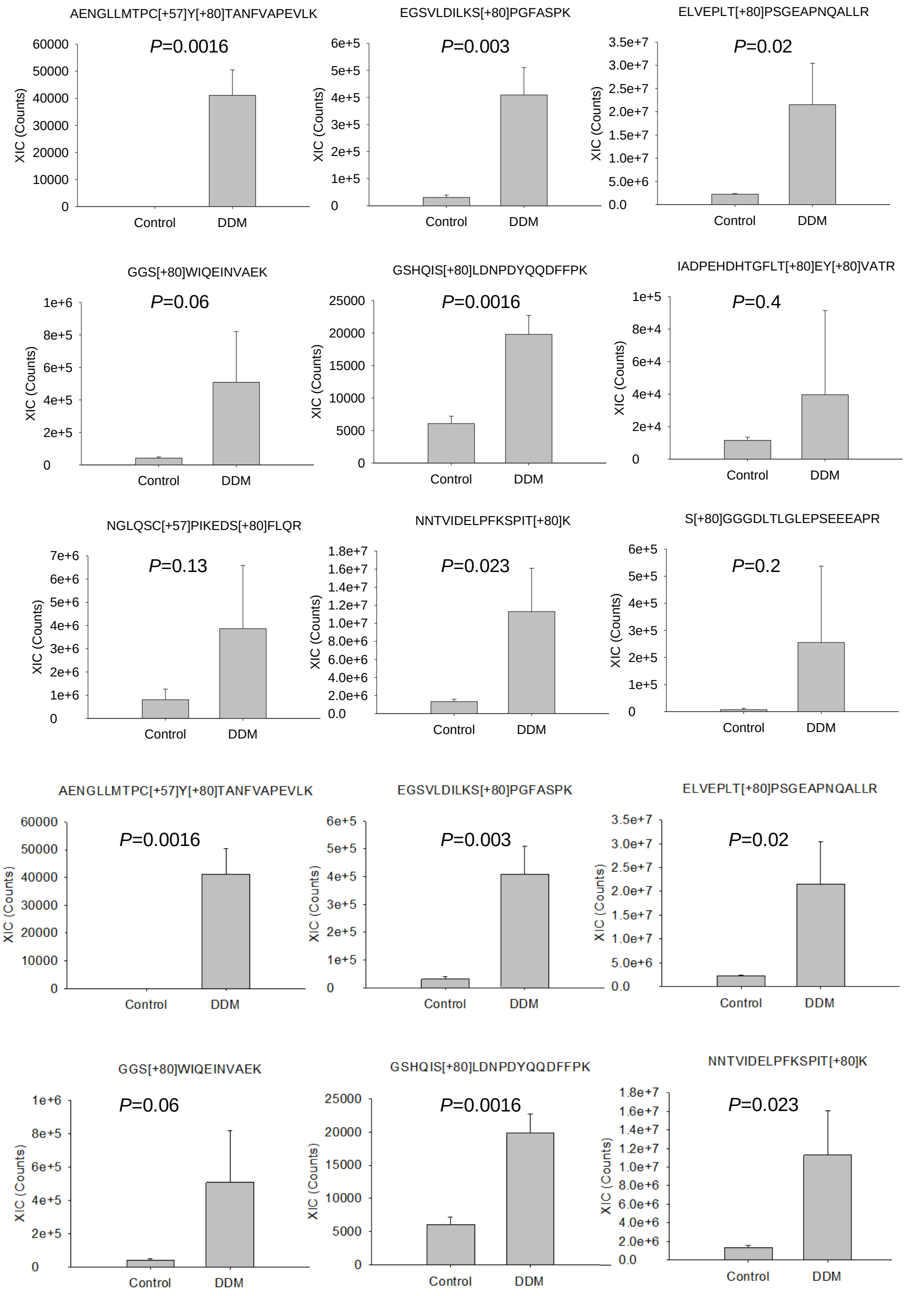

Supplementary Fig. 1 Evaluation of sample recovery and processing reproducibility in single PCR tube with and without DDM. SRM-based targeted quantification of a mixture of heavy isotope-labeled phosphopeptide standards without DDM (Control) and with DDM (DDM). XIC (Counts) corresponds to the SRM signal for peptide standards. The P values were shown for each peptide between without and with DDM additive.

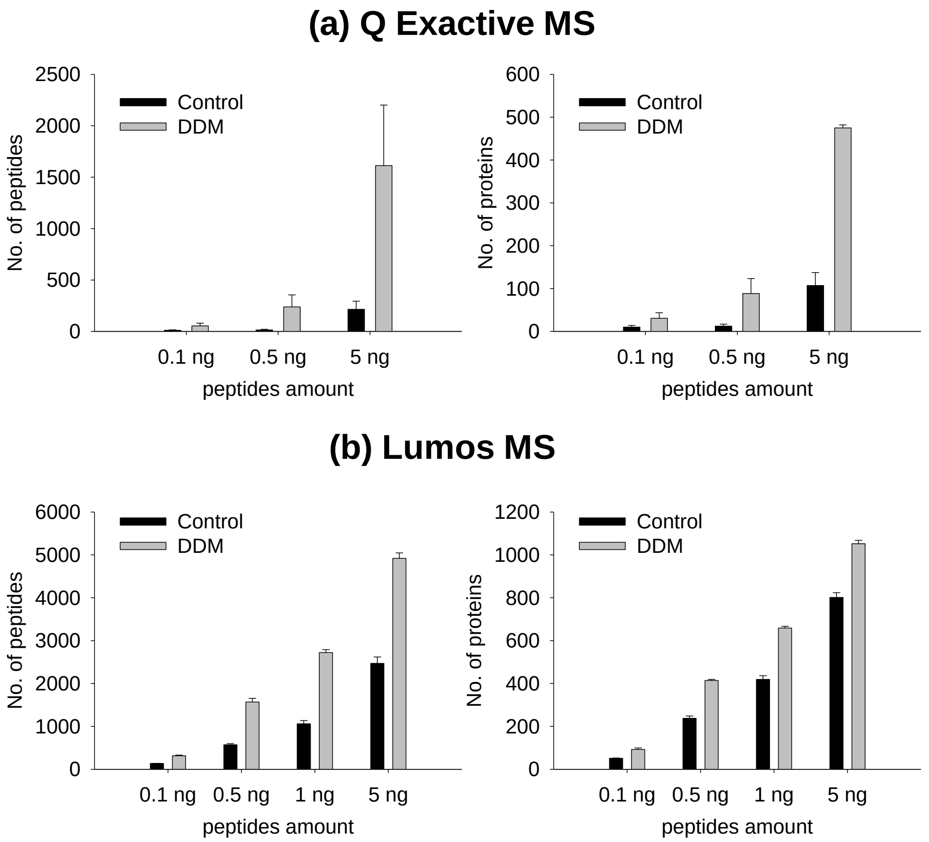

Supplementary Fig. 2 Number of unique peptides and protein groups identified by MS/MS only for 0.1, 0.5, 1, 5 ng of tryptic peptides from lung cancer PC9 cell lysate digests (equivalent to 1, 5, 10, and 50 cells) between without and with 0.015% DDM. Clearly, DDM can significantly improve the number of identified peptides and proteins (three biological replicates per condition). The data have been newly generated by two different groups using two different MS instruments (low-end Q Exactive MS and the most advanced Lumos MS).

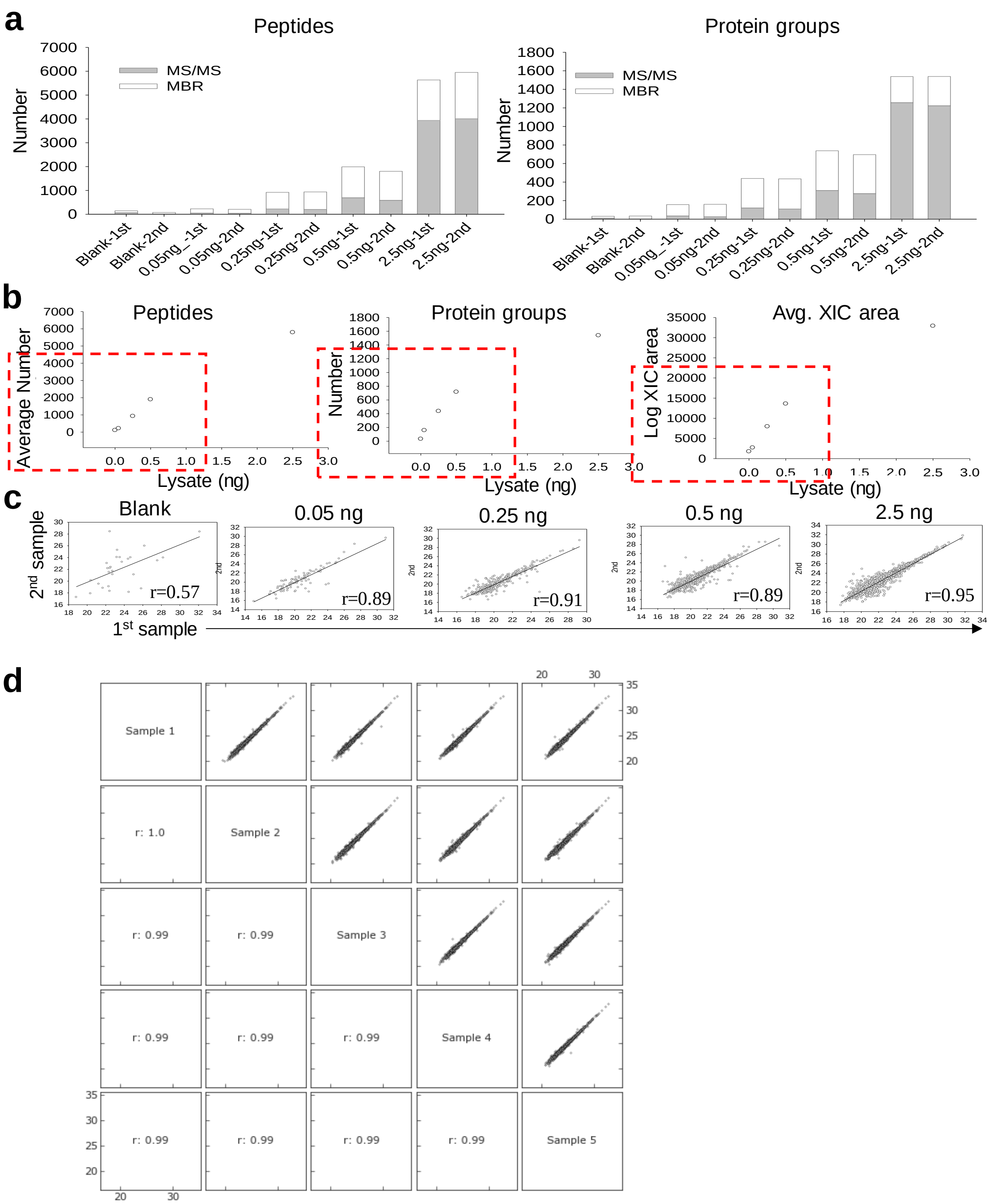

Supplementary Fig. 3 Evaluation of SOP-MS performance for analysis of low mass inputs of MCF7 cell lysates (0-2.5 ng) from serial dilution. a. Number of unique peptides (left panel) and protein groups (right panel) identified from 0, 0.05, 0.25, 0.5 and 2.5 ng of MCF7 lysates with duplicates for each mass input. **b.** The number of unique peptides, protein groups, and Log2 extracted ion chromatogram (XIC) area as a function of low mass inputs from MCF7 cell lysates (0, 0.05 ng≈0.5 cells, 0.25 ng≈2.5 cells, 0.5 ng≈5 cells). **c**. Correlations of Log_2LFQ between duplicates for each mass input. **d.** Correlations of Log_2LFQ between any two replicates out of 5 replicates for 5 ng of MCF7 cell lysates.

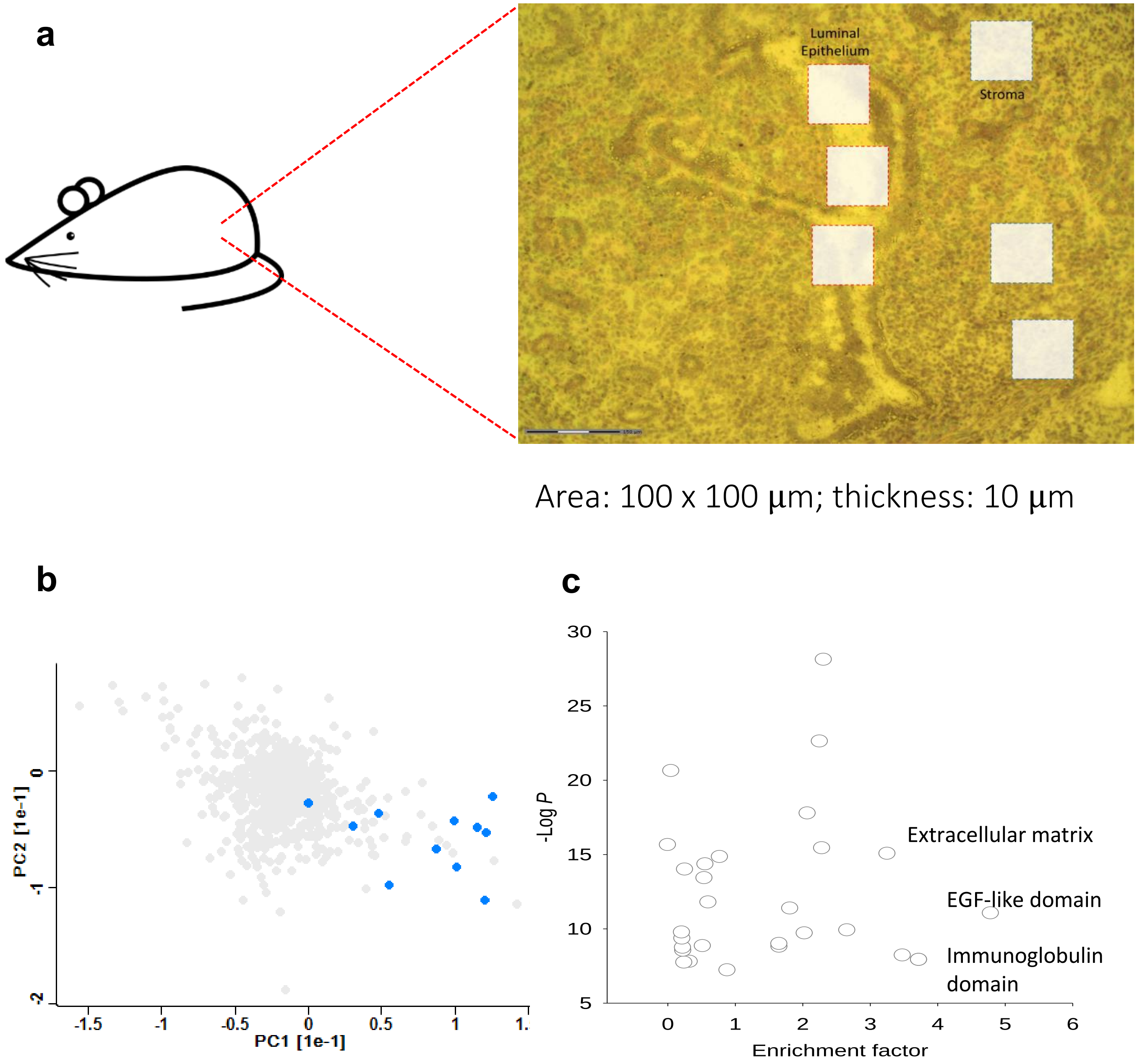

Supplementary Fig. 4 LCM dissected small sections from mouse uterine tissues. a. Image of three biological replicates for each tissue region (luminal epithelia and stroma) with a size of 100 μm in diameter and 10 μm in thickness (equivalent to ~20 cells). **b.** PCA analysis for identification of cell type-specific proteins. Blue dots indicate the proteins relevant to extracellular matrix receptor interactions and cell adhesion, which are specific to stroma region. **c.** Enriched proteins in the luminal epithelial region are relevant to EGF-like domain, immunoglobulin domain, and transmembrane domain.

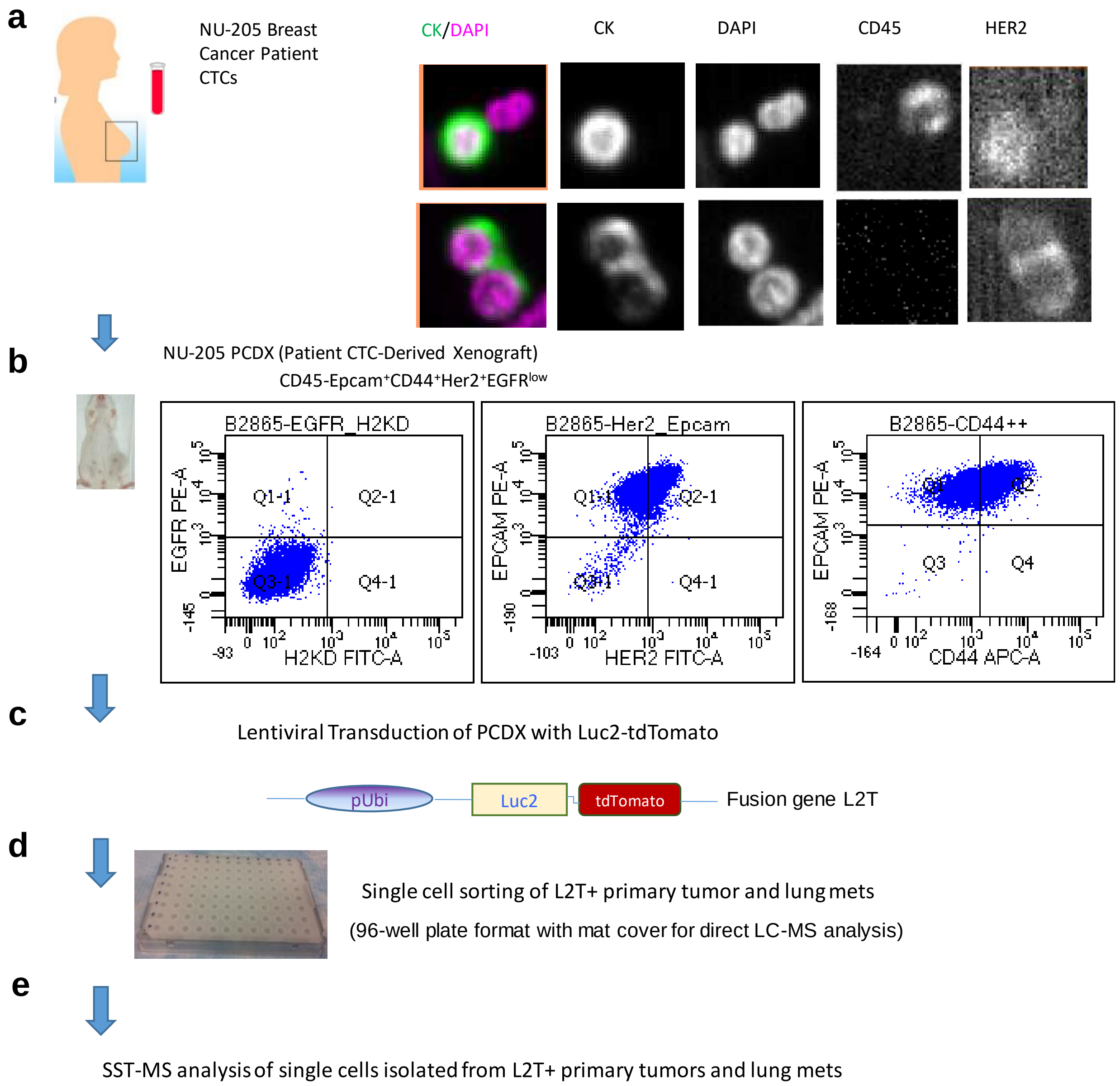

Supplementary Fig. 5 CTC-205 PDX model workflow a. CTCs isolated from blood of a breast cancer patient (NU-205) were confirmed to express cytokeratin (CK) and HER2 and to be negative for CD45 using the CellSearch platform analysis. **b.** CTCs from a breast cancer patient (NU-205) were enriched by depletion of $CD45^+$ PBMCs and implanted into NSG mouse mammary fat pads to generate the breast tumor xenograft PCDX-205. Flow cytometry profiles of the PCDX-205 show a negative expression of mouse stroma marker $H2K^d$ and proportional positive expression for human epithelial tumor markers EpCAM, HER2, CD44 and EGFR. **c.** PCDX-205 cells were transduced with lentivirus to express fluorescent L2T, and re-implanted in NSG mice. **d.** Tumors and lungs of PCDX-205-bearing mice were dissociated, and $L2T^+$ single cells from the tumor and lungs were sorted into a 96-well PCR plate. Cells were sorted based on $tdTomato^+$ expression. **e.** Single cells were analyzed by SOP-MS.

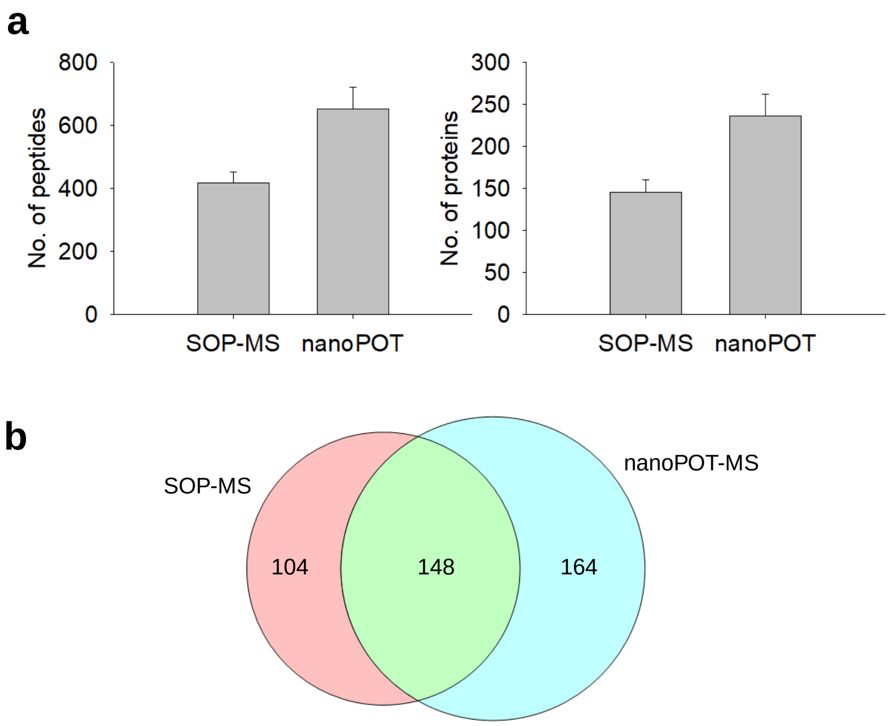

Supplementary Fig. 6 Performance comparison of SOP-MS with nanoPOTS-MS for analysis of single MCF10A cells. **a**, Number of unique peptides and protein groups identified by the MS/MS spectra alone from 4 single MCF10A cells sorted by FACS for each method. **b**, Venn diagram showing the number of total protein groups identified from each method.

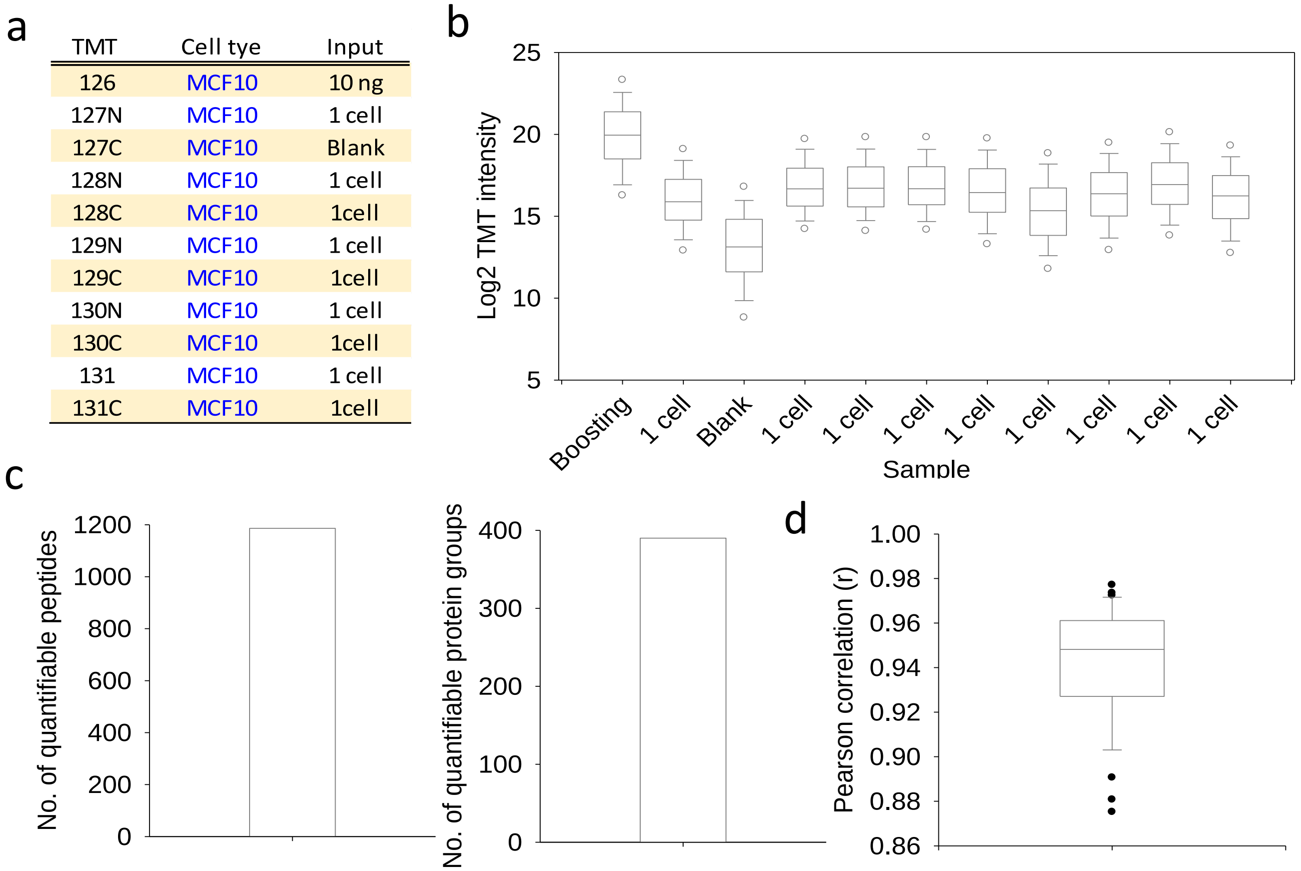

TMT	Cell tye	Input
126	MCF10	10 ng
127N	MCF10	1 cell
127C	MCF10	Blank
128N	MCF10	1 cell
128C	MCF10	1cell
129N	MCF10	1 cell
129C	MCF10	1cell
130N	MCF10	1 cell
130C	MCF10	1cell
131	MCF10	1 cell
131C	MCF10	1cell

Supplementary Fig. 7 Initial evaluation of multiplexed proteomic analysis of 9 single MCF10A cells sorted by FACS by using the combined TMT-based BASIL and SOP-MS. a. TMT-11 channel assignment (TMT-labeled sample channels for single MCF10A cells and a boosting channel with 10 ng of MCF10A cell lysate digests). **b**. Signal distribution of all TMT-11 channels. **c.** Number of quantifiable peptides and protein groups identified from single MCF10A cells. **d.** Pearson correlation for all 9 single MCF10A cells analyzed by SOP-MS with a median correlation of ~0.95. Half of sample injection was used for MS analysis (i.e., 0.5 single MCF10A cells for each channel).

Supplementary Table 1 Overview of current MS-based single-cell proteomics for label-free proteome profiling of regular-size single human cells.

**Single-cell processing method**	**nanoPOTS**			**iPAD-1**	**OAD**	**SOP**
**LC-MS setup**	LC flow rate: 60 nL/min<br>MS: Orbitrap Fusion Lumos	LC flow rate: 20 nL/min<br>MS: Orbitrap Fusion Lumos and Eclipse	LC flow rate: 60 nL/min<br>MS: Orbitrap Fusion Lumos	LC flow rate: 40 nL/min<br>MS: Orbitrap Fusion Lumos	LC flow rate: 200 nL/min<br>MS: Orbitrap MS	LC flow rate: 100 nL/min<br>MS: Q Exactive plus
**Single cells**	HeLa	HeLa	MCF10A	HeLa	HeLa	MCF10A
**MS/MS only**<br>*(The number of identified protein groups)*	211	362	236	128	51	146
**Device**	Chip-based microfluidic nanodroplet			In small i.d. (22 µm) capillary	Nanoliter-scale oil-air-droplet chip	PCR tube or multi-well plate
**Reference**	*Angew Chem, 130 (2018), 12550-12554*	*Anal Chem, 92 (2020), 2665-2671*	*Recent experiments (4 replicates)*	*Anal Chem, 90 (2018), 14003-14010*	*Anal Chem, 90 (2018), 5430-5438*	*Current work (4 replicates)*

Supplementary Materials and Methods

Stable isotope-labeled phosphopeptides. Crude stable isotope-labeled (SIL) phosphopeptides were synthesized with $^{13}C/^{15}N$ on C-terminal lysine or arginine (New England Peptide, Gardner, MA). The peptides were dissolved individually in 15% acetonitrile (ACN) and 0.1% formic acid (FA) at a concentration of 1.5 mM and stored at -80 °C. A mixture of these peptides was made with a final concentration of 10 pmol/µL for each peptide.

LC-SRM analysis. The SIL phosphopeptides were diluted by ddH_2O into 250 fmol/µL and analyzed using an Altis triple quadruple mass spectrometer (Thermo Fisher Scientific) equipped with a nanoACQUITY UPLC system (Waters, Milford, MA) for generating the data of **Supplementary Fig. 1**. Peptide samples were loaded onto an ACQUITY UPLC BEH 1.7-µm C18 column (100 µm i.d. × 10 cm). The mobile phases were (A) 0.1% FA in water and (B) 0.1% FA in ACN. 2 µL of the sample was loaded onto the column and separated at a flow rate of 400 nL/min using a 72-min gradient as followed (min:%B): 11:0.5, 13.5:10, 17:15, 38:25, 49:38, 50:95, 59:10, 60:95, 64:0.5. The LC column is operated at a temperature of 45 ºC. The parameters of the instrument were set as follows: Q1 and Q3 resolution were 0.7 fwhm, with 1 s cycle time. Data were acquired in scheduled SRM mode.

The selection of surrogate peptides for epidermal growth factor receptor (EGFR) pathway proteins and the SRM assays were described previously[1]. High-purity light peptides (>95%) were used to calibrate crude heavy peptide concentrations. Crude heavy isotope-labeled EGFR pathway peptide standards at a total amount of 30 fmol for each peptide were used for evaluation of peptide recovery with and without DDM (**Supplementary Table 1**). Samples were analyzed using a nanoACQUITY UPLC (Waters Corporation, Milford, MA) coupled to a TSQ Vantage triple quadrupole mass spectrometer (Thermo Scientific, San Jose, CA). The UPLC's nanoACQUITY UPLC BEH 1.7 µm C18 column (75 µm i.d. × 20 cm) was connected to a chemically etched 20 µm i.d. fused-silica electrospray emitter via a stainless metal union. Solvents used were 0.1% formic acid in water (mobile phase A) and 0.1% formic acid in 90% acetonitrile (mobile phase B). An amount of ~12 µL out of the total ~15 µL peptide sample was directly loaded onto the BEH C18 column from the PCR tube without using a trapping column. Sample loading and separation were performed at a flow rate of 350 and 300 nL/min, respectively. The binary LC gradient was used: 5-20% B in 26 min, 20-25% B in 10 min, 25-40% B in 8 min, 40-95% B in 1 min and at 95% B for 7 min for a total of 52 min, and the analytical column was re-equilibrated at 99.5% A for 8 min. The TSQ Vantage mass spectrometer was operated with ion spray voltages of 2400 ± 100 V, a capillary offset voltage of 35 V, a skimmer offset voltage of −5 V, and a capillary inlet temperature of 220 °C. The tube lens voltages were obtained from automatic tuning and calibration without further optimization. The retention time scheduled SRM mode was applied for SRM data collection with the scan window of ≥6 min. The cycle time was set to 1 s, and the dwell time for each transition was automatically adjusted depending on the number of transitions scanned at different retention time windows. A minimal dwell time 10 ms was used for each SRM transition. All the EGFR pathway proteins were simultaneously monitored in a single LC-SRM analysis.

Data analysis. Skyline software was used for all SRM data analysis[2]. The raw data were initially imported into Skyline software for visualization of chromatograms of target peptides to determine the detectability of target peptides. For each peptide the best transition without matrix

interference was used for precise quantification. Two criteria were used to determine the peak detection and integration: (1) same retention time and (2) approximately the same relative SRM peak intensity ratios across multiple transitions between endogenous (light) peptide and heavy peptide internal standards. All the data were manually inspected to ensure correct peak detection and accurate integration. The RAW data from TSQ Vantage were loaded into Skyline software to display graphs of extracted ion chromatograms (XICs) of multiple transitions of target proteins monitored.

Background of a PCDX model. In the dissemination of metastatic tumors, cancer cells from the primary tumor are shed into the peripheral blood vasculature. These circulating tumor cells (CTCs) serve as the vehicle by which primary tumors can seed distant metastases. In order to become a CTC, cancer cells from the primary tumor must undergo several steps to reach the bloodstream. Initially, tumor cells may undergo an epithelial to mesenchymal transition (EMT) and begin invading the surrounding extracellular matrix and basement membrane[3-5]. Eventually tumor cells will reach a local blood vessel and intravasate[6]. CTCs remain in the blood stream for up to several hours as single cells or clusters, sometimes associating with various other cell types, until they extravasate at a potential site of metastasis[7-10]. However, even in patients with advanced metastatic cancers, CTCs are a rare population (normally less than 0.1%) compared to peripheral blood mononuclear cells (PBMCs) within the blood. CTCs are commonly distinguished from other cell populations in the blood by negative expression of CD45, a leukocyte marker, and the positive expression of epithelial markers including EpCAM, cytokeratin, and/or other tumor associated antigens[11], which might be heterogeneous and not expressed in all CTCs. There remains understudied concerning the dynamic changes CTCs may undergo compared to tumor cells within the primary tumor and distant metastases. . Most notably, CTCs may exhibit cellular junction proteins and properties of cancer stem cells, which promote their ability to cluster and survive in the blood stream and seed distant metastases[12-16]. The detection of CTCs in singles and clusters in patient samples has shown important prognostic value[7, 14-17]. The characterization of CTC heterogeneity has been impeded due to the difficult sampling and maintenance of this rare population of tumor cells.

The development of patient derived xenografts (PDXs) that develop spontaneous metastases in mice has afforded researchers a representative model system to investigate the molecular and cellular basis of metastasis *in vivo* [13, 18]. In this study, we further established patient CTC-derived xenografts (PCDXs) which developed spontaneous lung metastasis, first creation to our knowledge, for single cell proteomic profiling of primary tumor cells as well as spontaneous lung metastases. Lentiviral labeling of this PCDX with the luciferase 2-tdTomato (L2T) dual fusion gene reporter enabled a convenient isolation and FACS-based single cell sorting of $L2T^+$ tumor cells from both primary tumor and lung metastasis after dissociation. The single cell proteomic profiling of PCDX model with metastasis not only allowed for the identification of new markers that can be leveraged for CTC isolation, but also facilitated elucidating the heterogeneous alterations of metastatic tumor cells upon colonization of the lungs.

Procedure for prioritization of the 18 differentially expressed proteins and generation of the heatmap: 1) After label-free quantification with MaxQuant MBR, the extracted ion chromatogram (XIC) areas of the identified protein groups were log2 transformed, and then normalized by the median value of each column; 2) The proteins containing at least 50% valid values in one group were kept in the data matrix, and the missing values were imputed by the normal distribution in

each column with a width of 0.3 and a downshift of 1.8 by using Perseus (Version 1.6.2.1); 3) The non-supervised PCA analysis was then used to generate PCA plot; 4) We further used Anova t-test to prioritize significantly differentiated proteins between lung metastatic and primary tumor cells ($p<0.05$, FDR<0.2) for the heatmap generation.

Supplementary References

1. Shi, T. et al. Conservation of protein abundance patterns reveals the regulatory architecture of the EGFR-MAPK pathway. *Sci Signal* **9**, rs6 (2016).
2. MacLean, B. et al. Skyline: an open source document editor for creating and analyzing targeted proteomics experiments. *Bioinformatics* **26**, 966-968 (2010).
3. Mani, S.A. et al. The epithelial-mesenchymal transition generates cells with properties of stem cells. *Cell* **133**, 704-715 (2008).
4. Wang, Y. et al. Vimentin expression in circulating tumor cells (CTCs) associated with liver metastases predicts poor progression-free survival in patients with advanced lung cancer. *Journal of Cancer Research and Clinical Oncology* (2019).
5. Hanahan, D. & Weinberg, R.A. Hallmarks of cancer: the next generation. *Cell* **144**, 646-674 (2011).
6. Pantel, K. & Speicher, M.R. The biology of circulating tumor cells. *Oncogene* **35**, 1216-1224 (2016).
7. Cristofanilli, M. et al. Circulating Tumor Cells, Disease Progression, and Survival in Metastatic Breast Cancer. *New England Journal of Medicine* **351**, 781-791 (2004).
8. Mu, Z. et al. Prospective assessment of the prognostic value of circulating tumor cells and their clusters in patients with advanced-stage breast cancer. *Breast Cancer Research and Treatment* **154**, 563-571 (2015).
9. Meng, S. et al. Circulating Tumor Cells in Patients with Breast Cancer Dormancy. *Clinical Cancer Research* **10**, 8152-8162 (2004).
10. Hong, Y., Fang, F. & Zhang, Q. Circulating tumor cell clusters: What we know and what we expect (Review). *Int J Oncol* **49**, 2206-2216 (2016).
11. Paoletti, C. & Hayes, D.F. in Novel Biomarkers in the Continuum of Breast Cancer. (ed. V. Stearns) 235-258 (Springer International Publishing, Cham; 2016).
12. Kreso, A. & Dick, John E. Evolution of the Cancer Stem Cell Model. *Cell Stem Cell* **14**, 275-291 (2014).
13. Liu, H. et al. Cancer stem cells from human breast tumors are involved in spontaneous metastases in orthotopic mouse models. *Proc Natl Acad Sci U S A* **107**, 18115-18120 (2010).
14. Liu, X. et al. Homophilic CD44 Interactions Mediate Tumor Cell Aggregation and Polyclonal Metastasis in Patient-Derived Breast Cancer Models. *Cancer Discov* **9**, 96-113 (2019).
15. Aceto, N. et al. Circulating tumor cell clusters are oligoclonal precursors of breast cancer metastasis. *Cell* **158**, 1110-1122 (2014).
16. Gkountela, S. et al. Circulating Tumor Cell Clustering Shapes DNA Methylation to Enable Metastasis Seeding. *Cell* **176**, 98-112 e114 (2019).
17. Alix-Panabières, C. & Pantel, K. Clinical Applications of Circulating Tumor Cells and Circulating Tumor DNA as Liquid Biopsy. *Cancer Discovery* **6**, 479-491 (2016).
18. Liu, H. et al. Cancer stem cells from human breast tumors are involved in spontaneous metastases in orthotopic mouse models. *Proc Natl Acad Sci U S A* **107**, 18115-18120 (2010).
