# Supplementary material for: Surfactant-assisted one-pot sample preparation for label-free single-cell proteomics
Source: Commun Biol. 2021 Mar 1;4:265. doi: 10.1038/s42003-021-01797-9 (PMC7921383; doi:10.1038/s42003-021-01797-9)
Supplement: Supplementary file 2 — Description of Additional Supplementary Files [file 42003_2021_1797_MOESM2_ESM.pdf]

**Legends for Supplementary Data 1-4:**

**Supplementary Data 1**
**Description:** SRM-based targeted quantification of heavy isotope-labeled EGFR pathway peptide standards (the best transition without interference) at different concentrations of DDM.

**Supplementary Data 2**
**Description:** Proteins with a significant difference in abundance between two uterine tissue subregions by label-free quantification.

**Supplementary Data 3**
**Description:** Proteins with a significant difference in abundance between two types of single cells (i.e., 10 primary tumor vs 10 lung metastasis) by label-free quantification.

**Supplementary Data 4**
**Description:** The index of all RAW data files for the main figures.
