# Supplementary material for: Surfactant-assisted one-pot sample preparation for label-free single-cell proteomics
Source: Commun Biol. 2021 Mar 1;4:265. doi: 10.1038/s42003-021-01797-9 (PMC7921383; doi:10.1038/s42003-021-01797-9)
Supplement: Supplementary file 3 — Reporting Summary [file 42003_2021_1797_MOESM3_ESM.pdf]

nature research

Corresponding author(s): Tujin Shi, Huiping Liu, Tao Liu

Last updated by author(s): Dec 1, 2020

Reporting Summary

Nature Research wishes to improve the reproducibility of the work that we publish. This form provides structure for consistency and transparency in reporting. For further information on Nature Research policies, see Authors & Referees and the Editorial Policy Checklist.

Statistics

For all statistical analyses, confirm that the following items are present in the figure legend, table legend, main text, or Methods section.

n/a	Confirmed	
☐	☒	The exact sample size (n) for each experimental group/condition, given as a discrete number and unit of measurement
☐	☒	A statement on whether measurements were taken from distinct samples or whether the same sample was measured repeatedly
☐	☒	The statistical test(s) used AND whether they are one- or two-sided<br>*Only common tests should be described solely by name; describe more complex techniques in the Methods section.*
☒	☐	A description of all covariates tested
☒	☐	A description of any assumptions or corrections, such as tests of normality and adjustment for multiple comparisons
☐	☒	A full description of the statistical parameters including central tendency (e.g. means) or other basic estimates (e.g. regression coefficient) AND variation (e.g. standard deviation) or associated estimates of uncertainty (e.g. confidence intervals)
☒	☐	For null hypothesis testing, the test statistic (e.g. F, t, r) with confidence intervals, effect sizes, degrees of freedom and P value noted<br>*Give P values as exact values whenever suitable.*
☒	☐	For Bayesian analysis, information on the choice of priors and Markov chain Monte Carlo settings
☒	☐	For hierarchical and complex designs, identification of the appropriate level for tests and full reporting of outcomes
☒	☐	Estimates of effect sizes (e.g. Cohen's d, Pearson's r), indicating how they were calculated

Our web collection on statistics for biologists contains articles on many of the points above.

Software and code

Policy information about availability of computer code

Data collection	Commercial MS software Xcalibur was used to collect all RAW data.
Data analysis	The freely-available open-source MaxQuant software (Version 1.5.1.11) was used for protein identification and quantification. Biological functions and signaling pathways were analyzed by using DAVID Bioinformatics Resources (Version 6.8) and Peruses (Version 1.6.2.1), and protein-protein association network analysis was performed by the latest version of STRING (Version 11.0).

For manuscripts utilizing custom algorithms or software that are central to the research but not yet described in published literature, software must be made available to editors/reviewers. We strongly encourage code deposition in a community repository (e.g. GitHub). See the Nature Research guidelines for submitting code & software for further information.

Data

Policy information about availability of data

All manuscripts must include a data availability statement. This statement should provide the following information, where applicable:

- Accession codes, unique identifiers, or web links for publicly available datasets
- A list of figures that have associated raw data
- A description of any restrictions on data availability

The RAW global proteomics data and the identified protein groups from MaxQuant have been deposited in Japan ProteOme STandard Repository (jPOST: https://repository.jpostdb.org/). The accession codes: JPST000866 for jPOST and PXD019626 for ProteomeXchange. The Skyline-processed SRM results and the RAW targeted proteomics data can be accessed without restrictions at Panorama (Access link: https://panoramaweb.org/AMPFxF.url) and ProteomeXchange (Accession code: PXD022827), respectively.

Field-specific reporting

Please select the one below that is the best fit for your research. If you are not sure, read the appropriate sections before making your selection.

[x] Life sciences [ ] Behavioural & social sciences [ ] Ecological, evolutionary & environmental sciences

For a reference copy of the document with all sections, see nature.com/documents/nr-reporting-summary-flat.pdf

Life sciences study design

All studies must disclose on these points even when the disclosure is negative.

Sample size	No sample-size calculation was performed. Two types of single cells were used for method development and 20 single cells from a PCDX model were used to demonstrate broad utility of our method.
Data exclusions	No data were excluded.
Replication	All attempts at replication were successful. The method reproducibility was evaluated with two types of single cells with three or four biological replicates for each cell type.
Randomization	This is not relevant to our study. Two types of single cells were used for method development and 20 single cells from a PCDX model were used to demonstrate broad utility of our method. We do not target specific and comprehensive biological studies.
Blinding	Blinding was not relevant to our study. It is method development and demonstration.

Reporting for specific materials, systems and methods

We require information from authors about some types of materials, experimental systems and methods used in many studies. Here, indicate whether each material, system or method listed is relevant to your study. If you are not sure if a list item applies to your research, read the appropriate section before selecting a response.

Materials & experimental systems

n/a	Involved in the study	
[x]	[ ]	Antibodies
[x]	[ ]	Eukaryotic cell lines
[x]	[ ]	Palaeontology
[ ]	[x]	Animals and other organisms
[x]	[ ]	Human research participants
[x]	[ ]	Clinical data

Methods

n/a	Involved in the study	
[x]	[ ]	ChIP-seq
[x]	[ ]	Flow cytometry
[x]	[ ]	MRI-based neuroimaging

Animals and other organisms

Policy information about studies involving animals; ARRIVE guidelines recommended for reporting animal research

Laboratory animals	8-10 weeks old female NSG mice.
Wild animals	This is not relevant to our study.
Field-collected samples	8-10 weeks old female NSG mice were used for implantation of human breast cancer PCDX models and kept in specific pathogen-free facilities in the Animal Resources Center at Northwestern University.Breast tumors were harvested after 2-3 months and confirmed as a human PCDX with positive expression of human epithelial markers EpCAM, HER2, and CD44 as well as negative expression of mouse H2Kd.
Ethics oversight	Animal procedures and experimental procedures have been performed under approval by Northwestern University Animal Care and Use Committee (ACUC) and complied with the NIH Guidelines for the Care and Use of Laboratory Animals.

Note that full information on the approval of the study protocol must also be provided in the manuscript.
